# Development of an MRI contrast agent for both detection and inhibition of the amyloid-β fibrillation process†

**DOI:** 10.1039/d2ra00614f

**Published:** 2022-02-09

**Authors:** Rohmad Yudi Utomo, Satoshi Okada, Akira Sumiyoshi, Ichio Aoki, Hiroyuki Nakamura

**Affiliations:** School of Life Science and Technology, Tokyo Institute of Technology 4259 Nagatsuta, Midori Yokohama Kanagawa 226-8503 Japan; Laboratory for Chemistry and Life Science, Institute of Innovative Research, Tokyo Institute of Technology 4259 Nagatsuta, Midori Yokohama Kanagawa 226-8503 Japan sokada@res.titech.ac.jp hiro@res.titech.ac.jp; JST, PRESTO 4259 Nagatsuta, Midori Yokohama Kanagawa 226-8503 Japan; Institute for Quantum Medical Science, National Institutes for Quantum Science and Technology 4-9-1 Anagawa, Inage Chiba 263-8555 Japan

## Abstract

A curcumin derivative conjugated with Gd-DO3A (Gd-DO3A-Comp.B) was synthesised as an MRI contrast agent for detecting the amyloid-β (Aβ) fibrillation process. Gd-DO3A-Comp.B inhibited Aβ aggregation significantly and detected the fibril growth at 20 μM of Aβ with 10 μM of probe concentration by *T*_1_-weighted MR imaging.

## Abstract 2

A curcumin derivative conjugated with Gd-DO3A (Gd-DO3A-Comp.B) was developed to significantly inhibit the amyloid-β (Aβ) aggregation and detect the fibril growth by *T*_1_-weighted MR imaging.

## Graphical abstract

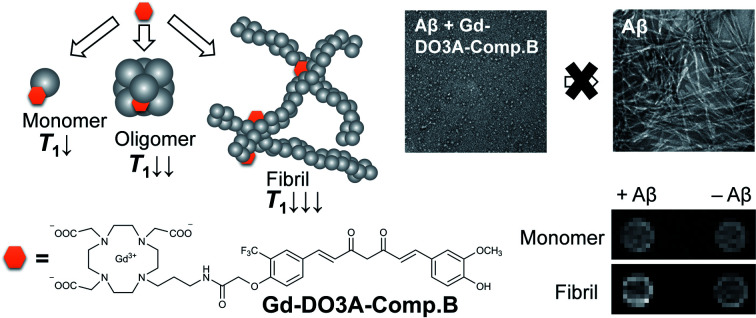

A significant increase of Alzheimer's disease (AD) patients urges the development of therapeutic and diagnostic technology.^1^ As with the therapeutic development, diagnostic technology also faces several obstacles. To date, the definite diagnosis of AD relies on the histopathological data of post-mortem.^2,3^ The non-invasive imaging technology targeting AD biomarkers such as amyloid β (Aβ) could provide phenotypical diagnostics, although the development of Aβ probes still remains challenging. Several contrast agents for single photon emission computed tomography (SPECT) and positron emission tomography (PET) such as Florbetapir-^18^F and Pittsburgh compound-B ([^11^C]PiB) were developed as efficient tracers in mild cognitive impairment patients.^4,5^ However, PET- and SPECT-based diagnostics require injection of radioactive probes, which cannot be measured frequently due to radiation exposure and limited availability of facilities. They also provide limited information on the anatomic profile of biomarkers due to their low spatial resolution and imprecise microscopic localization.^6^ In contrast, magnetic resonance imaging (MRI) contrast agents could quantify the Aβ accumulation in the anatomic brain image.^7^

Several reported MRI contrast agents using gadolinium (Gd) complexes demonstrate potential use of Aβ detection. A clinically approved contrast agent, Gd(iii) diethylenetriaminepentaacetic acid (Gd-DTPA) complex accumulates in brain after opening the blood–brain barrier (BBB) by using mannitol and detects Aβ deposits in the mice AD-model.^8^ To improve the selectivity, Gd complexes were conjugated with compounds binding to Aβ such as Pittsburgh compound B (Gd-DO3A-PiB) which also serves as an approach for increasing MRI sensitivity.^9,10^ An α,β-unsaturated ketone compound curcumin has been widely reported as an Aβ probe due to its ability to bind the hydrophobic site of Aβ.^11,12^ Allen *et al.* firstly reported the direct conjugation of curcumin with Gd-DTPA which binds to Aβ with four times higher relaxivity than free Gd-DTPA.^13^ Furthermore, a polymalic acid-based nanoparticle covalently linked with curcumin and Gd-DOTA could also detect Aβ in human brain specimen by MRI.^14^ These previous studies demonstrate that the curcumin structure has significant potential for the development of MRI contrast agents for AD diagnosis.

Previously, we reported a curcumin derivative, compound B, possesses 100-times stronger inhibitory activity of Aβ aggregation than curcumin on the basis of thioflavin T (ThT) competitive binding assay.^15,16^ According to this result, we designed curcumin-based Gd probes for the detection and inhibition of Aβ (Fig. 1A–C). We hypothesized that these probes could accelerate proton longitudinal relaxation depending on the fibrillation stage of Aβ, because molecular tumbling rate of the Gd complexes becomes slower (Fig. 1A).^17^ As a result, the probes permit the detection of Aβ by longitudinal relaxation time (*T*_1_)-weighted imaging. This mechanism could also be utilized to estimate the inhibitory activity of the probes by *T*_1_-based analysis (Fig. 1B). The curcumin and compound B were directly conjugated with the macrocyclic DO3A ligand through the propylamine linker to obtain Gd-DO3A-Cur and Gd-DO3A-Comp.B, respectively (Fig. 1C).

**Fig. 1:**
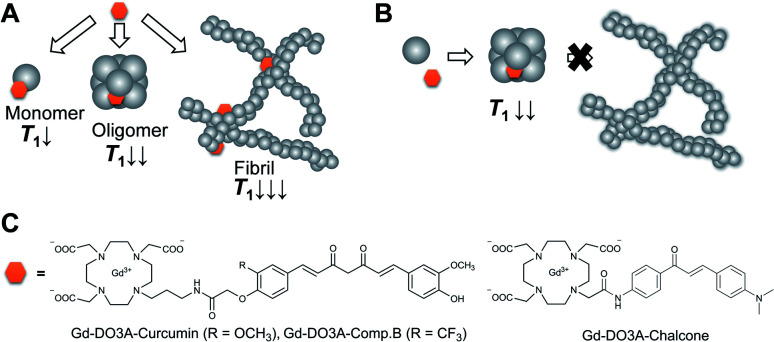
(A) A probe concept that produces *T*_1_ in a dependent manner of Aβ fibrillation process. (B) Inhibitor-based probes that cause moderate *T*_1_ decreases due to inhibitory activity of fibrillation. (C) The chemical structures of the synthesized Gd probes for Aβ detection and inhibition.

Gd-DO3A-Cur and Gd-DO3A-Comp.B were synthesized according to Scheme 1 (detail in Scheme S1, ESI†). The compound 5a and 5b, which have asymmetric curcumin derivatives containing carboxylic acid group, were synthesized by three step reactions. Amide bond formation with DO3A(*t*Bu)_3_-propylamine ligand^18^ by condensation reaction afforded compound 7a and 7b. The *tert*-butyl groups were deprotected by trifluoroacetic acid producing compound 8a and 8b. The complexation was performed with GdCl_3_·6H_2_O by adjusting the reaction pH to 7, giving 43 and 41% yields of Gd-DO3A-Cur and Gd-DO3A-Comp.B, respectively. The *T*_1_ relaxivities (*r*_1_) of the curcumin-based Gd probes were estimated by *T*_1_ measurement using a 1 tesla NMR relaxometry (Fig. S1, ESI†). For the comparison, we synthesized Gd-DO3A-Chal which is a reported probe for Aβ.^19^ The *r*_1_ of Gd-DO3A-Comp.B, Gd-DO3A-Cur, and Gd-DO3A-Chal were 7.1, 6.1 and 5.3 mM^−1^ s^−1^, respectively. These *r*_1_ values are higher than that of clinically approved Gd-DOTA (3.9 mM^−1^ s^−1^).^20^ The molecular weight of Gd-DO3A-Comp.B and Gd-DO3A-Cur is almost two times larger than that of Gd-DOTA. Because the *r*_1_ increases approximately linearly with molecular weight in low magnetic field,^17^ the high *r*_1_ values of Gd-DO3A-Comp.B and Gd-DO3A-Cur might be mainly attributed to their high rotational correlation time, rather than the high number of coordinated water molecules. The *r*_1_ of Gd-DO3A-Chal was comparable to the value reported previously.^19^

**Scheme 1:**
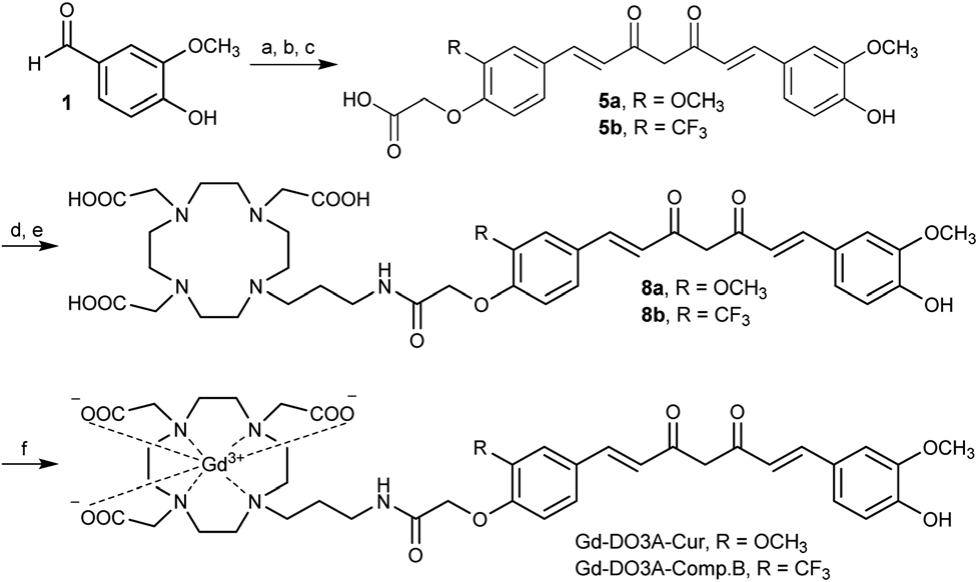
Synthetic scheme of Gd-DO3A-Cur and Gd-DO3A-Comp.B. (a) B(OH)_3_, morpholine, DMF, 100 °C, 10 min. (b) 3a/3b, B(OH)_3_, morpholine, DMF, 100 °C, 10 min. (c) TFA, DCM. (d) DO3A(*t*Bu)_3_-propylamine ligand, PyBOP, HOBt, Et_3_N, DMF. (e) 7a/7b, TFA, DCM. (f) GdCl_3_·6H_2_O, NaOH, H_2_O.

We evaluated the inhibitory effect of three probes toward Aβ aggregation by Congo red assay.^21^ After 24 h incubation of 20 μM Aβ with 10 μM probe, Gd-DO3A-Comp.B showed the lowest fluorescence intensity, indicating the strongest inhibitory activity followed by Gd-DO3A-Cur (Fig. 2A). As the comparison, the reported MRI agents, Gd-DO3A-Chal showed slight inhibitory activity. The inhibitory effect was further evaluated by transmission electron microscopy (TEM) with negative staining (Fig. 2B). In the absence of the probes, Aβ formed huge and massive fibril similar to the typical morphology of Aβ fibril.^22^ The TEM images of Aβ with Gd-DO3A-Comp.B showed the presence of white spheres below 10 nm, demonstrating that Gd-DO3A-Comp.B strongly inhibits Aβ aggregation. In fact, the fibril growth stopped at a stage of oligomer formation. Lower inhibitory activity of Gd-DO3A-Cur was also found to provide a shortened worm-like fibril, which is the typical morphology of Aβ exposed to curcumin.^23^ In contrast, the small amount of white spheres and partial fibril disruption were found in the image of Aβ with Gd-DO3A-Chal. In comparison with a reported Gd-DTPA-curcumin possessing inhibitory activity starting at 50 μM, Gd-DO3A-Comp.B possessed stronger inhibition of Aβ aggregation at 10 μM.^24^ The MTT assay using Neuro 2a cells showed that IC_50_ of Gd-DO3A-Cur and Gd-DO3A-Comp.B. were more than 500 μM, indicating that these compounds did not possess significant cytotoxicity (Fig. S2, ESI†).

**Fig. 2:**
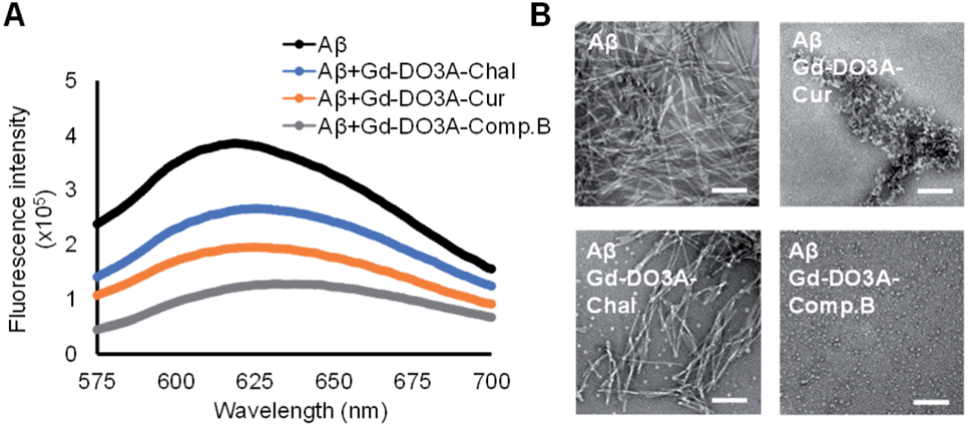
Inhibitory effect of the Gd probes toward Aβ aggregation measured by Congo red assay (A) and negative staining TEM images (B). The Gd probes were co-incubated with monomeric Aβ for 24 h in PBS at pH 7.4. [Gd] = 10 μM, [Aβ] = 20 μM. Scale bars = 100 nm.

To detect fibrillation process by NMR relaxometry, we measured *T*_1_ of the probe mixture with Aβ which were pre-incubated for 1, 3, 6, 12, and 24 h to make it form the fibrils of different growth stages (Fig. 3A and B). The *T*_1_ of Gd-DO3A-Comp.B solution decreased with pre-incubation time of Aβ, demonstrating that the Gd-DO3A-Comp.B can detect Aβ fibril depending on the growth stage (Fig. 3B). Lower *T*_1_ involved with Aβ growth could be caused by the reduction in tumbling rate of the Gd complex.^25^ We also co-incubated the probes with the Aβ monomer and monitored *T*_1_ changes over the incubation time (Fig. 3A, B and S3, ESI†). Interestingly, the Gd-DO3A-Comp.B did not cause significant *T*_1_ decreases even after 24 hours co-incubation with Aβ monomers, demonstrating that Gd-DO3A-Comp.B has a strong inhibitory effect on fibril formation and the inhibition can be monitored by *T*_1_ measurement (Fig. 3B). The inhibitory effect was consistent with the results of Congo red assay and TEM (Fig. 2). On the other hand, the time-dependent increases of *T*_1_ were observed in Gd-DO3A-Chal and Gd-DO3A-Cur. This might be because these two probes were buried in the hydrophobic pocket as Aβ fibril grew up and fewer water molecules permitted access to the Gd ions. It is also possible that these probes have lower binding affinity, especially for matured fibril, and require higher concentrations to produce significant *T*_1_ changes.^26^ These probe did not produce the significant Δ*T*_1_ between monomer and fibril samples (Fig. 3B and S3, ESI†), although they showed little inhibition in Congo red assay and TEM (Fig. 2).

**Fig. 3:**
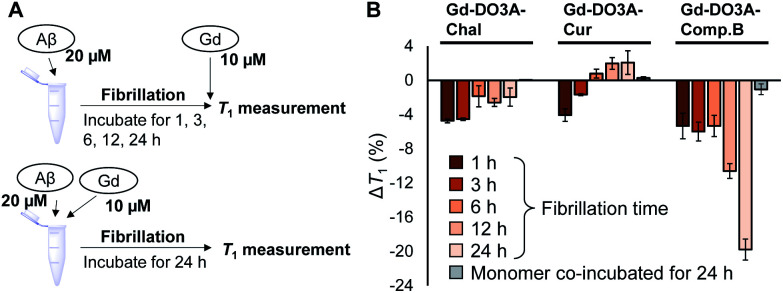
(A) Experimental design of *T*_1_-based detection of Aβ fibrillation and inhibition by using the Gd probes. (B) *T*_1_ changes of the Gd probe solutions with pre-incubated fibrils and monomers in PBS at pH 7.4 (mean ± SEM, *n* = 3). [Gd] = 10 μM, [Aβ] = 20 μM.

The feasibility of the Gd probes was further evaluated by *in vitro* MRI measurement using a 1 tesla scanner. The *T*_1_-weighted images showed that Gd-DO3A-Comp.B produced slight *T*_1_ signal increases with Aβ monomers for 2 and 24 h (Fig. 4A and B). More significant signal increases were observed in the Gd-DO3A-Comp.B with Aβ fibril pre-incubated for 24 h (Fig. 4C). In contrast, Gd-DO3A-Chal and Gd-DO3A-Cur did not show significant signal changes in the presence of Aβ monomers or fibrils (Fig. 4A–C). These results were mostly consistent with the *T*_1_ profile measured by NMR (Fig. 3). Compared to the previously reported Gd-DO3A-Chal that required 100 μM of the probe concentration to detect the equimolar Aβ,^19^ Gd-DO3A-Comp.B could detect five-times lower concentration of Aβ (20 μM) with ten-times lower probe concentration (10 μM). Therefore, Gd-DO3A-Comp.B could be promising to further develop highly sensitive diagnostic MRI contrast agents of AD.

**Fig. 4:**
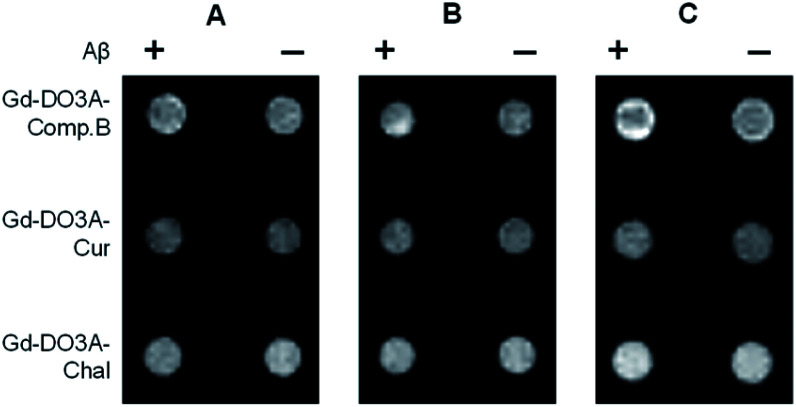
*T*_1_-weighted images of the Gd probe solutions in the presence of monomeric Aβ at 2 h incubation (A), monomeric Aβ at 24 h incubation (B), and Aβ fibrils pre-incubated for 24 h (C). Incubation was conducted in PBS at pH 7.4.

In conclusion, we synthesized the curcumin-based Gd probes which enabled the detection and inhibition of Aβ fibril formation. Gd-DO3A-Comp.B allowed for the highly sensitive detection of Aβ fibril by the *T*_1_ measurement. Moreover, the inhibitory activity could be estimated by *T*_1_ measurement, because Gd-DO3A-Comp.B decreased *T*_1_ depending on the growth stage of Aβ fibril formation. Such unique modality would be useful not only for the diagnostics but also for the direct evaluation of the therapeutic efficacy *in vivo*. For the future application, it would be important to combine with BBB penetration methods targeting the brain such as transient opening of the BBB using focused ultrasound or mannitol injection.^27,28^

## Conflicts of interest

There are no conflicts to declare.

## Supplementary Material

RA-012-D2RA00614F-s001
