# Supplementary material for: Development of an MRI contrast agent for both detection and inhibition of the amyloid-β fibrillation process
Source: RSC Adv. 2022 Feb 9;12(8):5027–30. doi: 10.1039/d2ra00614f (PMC8981495; doi:10.1039/d2ra00614f)

Electronic Supplementary Material (ESI) for RSC Advances.
This journal is © The Royal Society of Chemistry 2022

Supplementary Information

Development of an MRI contrast agent for both detection and inhibition of the amyloid-β fibrillation process

Rohmad Yudi Utomo,[a] Satoshi Okada,[a,b,c*] Akira Sumiyoshi,[d] Ichio Aoki,[d] Hiroyuki Nakamura[a,b*]

[a]*School of Life Science and Technology, Tokyo Institute of Technology, 4259 Nagatsuta, Midori, Yokohama, Kanagawa 226-8503, Japan*
[b]*Laboratory for Chemistry and Life Science, Institute of Innovative Research, Tokyo Institute of Technology, 4259 Nagatsuta, Midori, Yokohama, Kanagawa 226-8503, Japan*
[c]*JST, PRESTO, 4259 Nagatsuta, Midori, Yokohama, Kanagawa 226-8503, Japan.*
[d]*Institute for Quantum Medical Science, National Institutes for Quantum and Radiological Science and Technology, 4-9-1 Anagawa, Inage, Chiba, Chiba 263-8555, Japan*

Email: Satoshi Okada (sokada@res.titech.ac.jp); Hiroyuki Nakamura (hiro@res.titech.ac.jp)

Table of Content

Scheme S1………………………………………………………………………. S2

Figure S1………………………………………………………………………. S2

Figure S2………………………………………………………………………. S3

Figure S3………………………………………………………………………. S3

Figure S4………………………………………………………………………. S4

Experimental Procedure………………………………………………………. S5

References……………………………………………………………………. S10

^{1}H, ^{13}C, and ^{19}F NMR Spectra of Compounds …………………………….. S11-S25

Purity Analysis of Final Compounds……………………………………….. S26

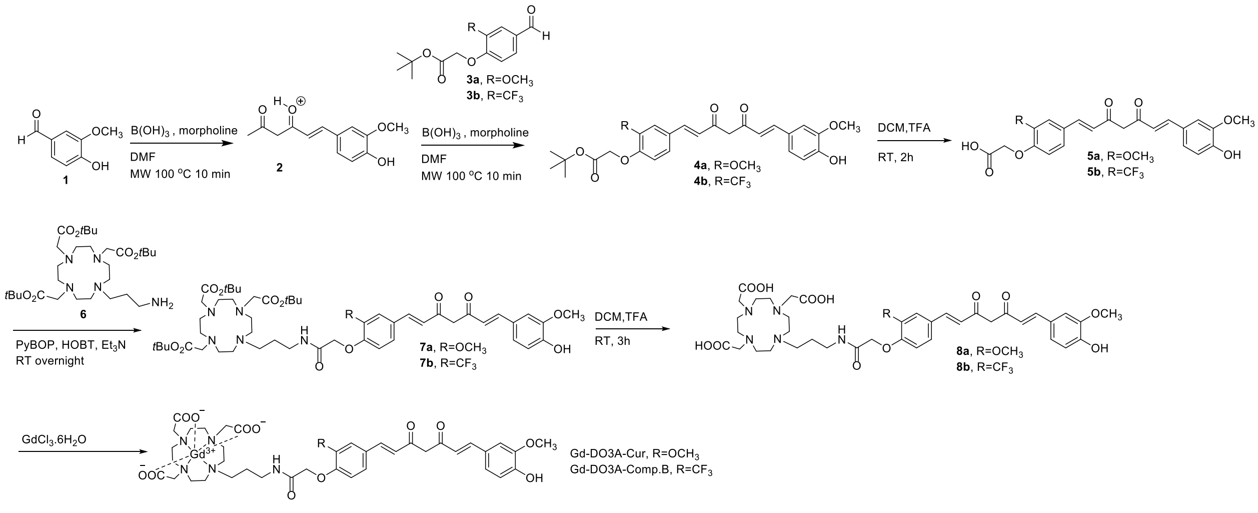

Scheme S1. Synthetic scheme of curcumin derivatives.

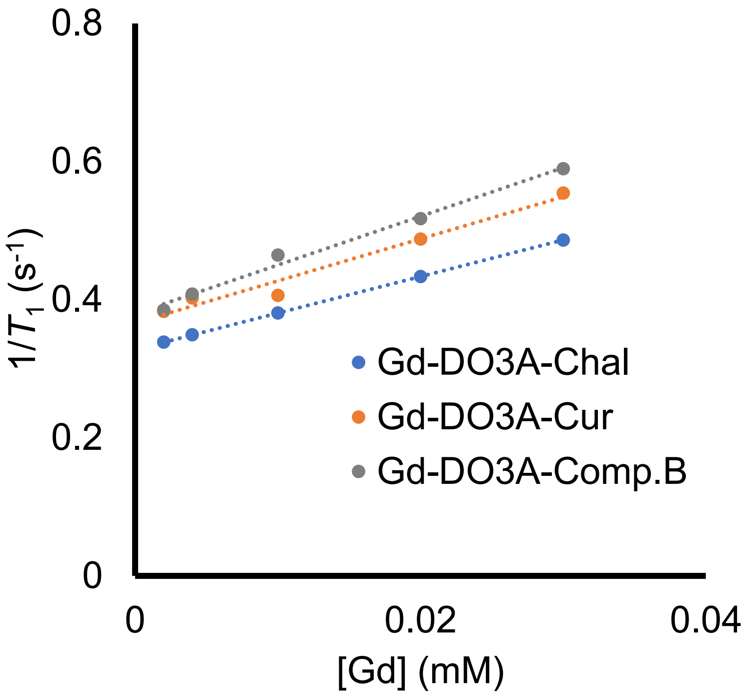

Figure S1. Relaxation rate profile of the Gd probes.

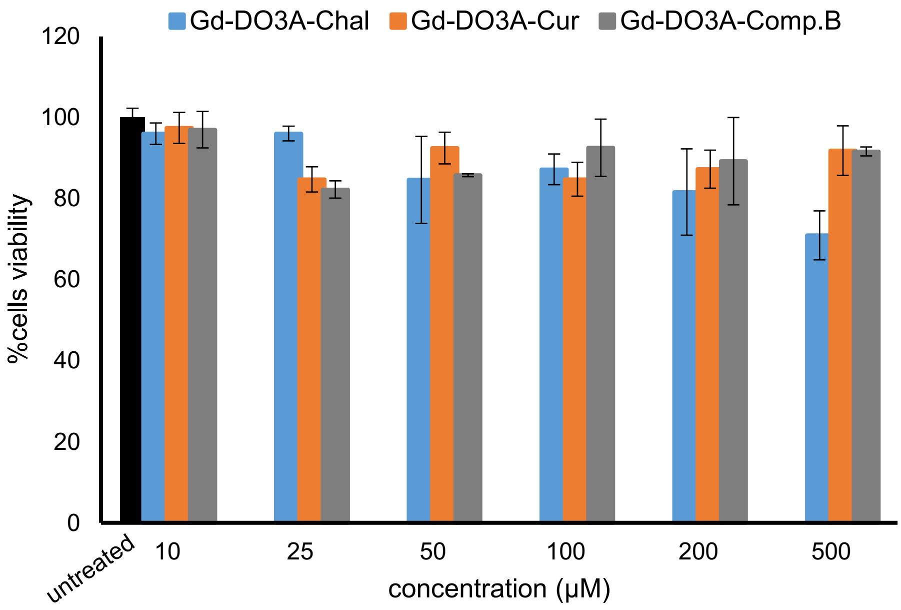

Figure S2. Cytotoxicity study of Gd compounds on Neuro 2a cells

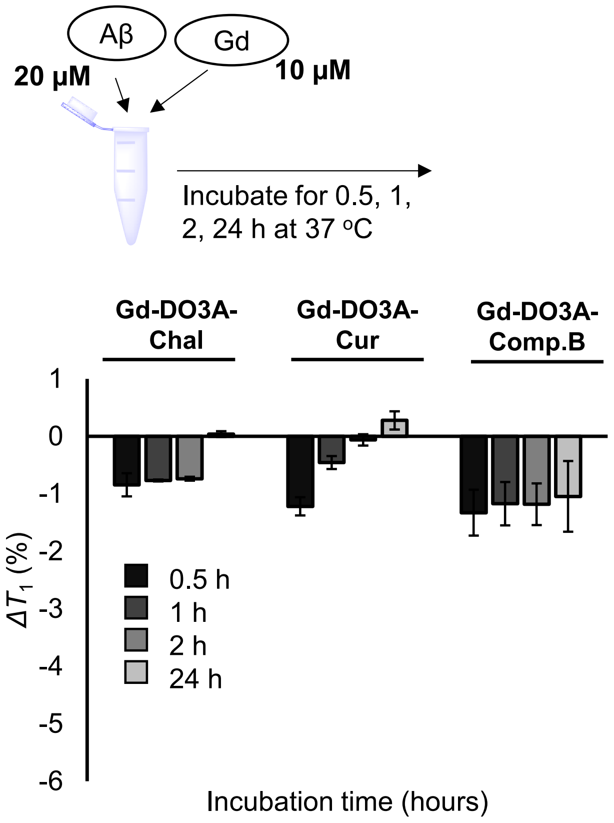

Figure S3. T_1 changes of Gd-DO3A-Chal, Gd-DO3A-Cur, and Gd-DO3A-Comp.B co-incubated with Aβ monomer for 0.5, 1, 2, and 24 h at 37 °C.

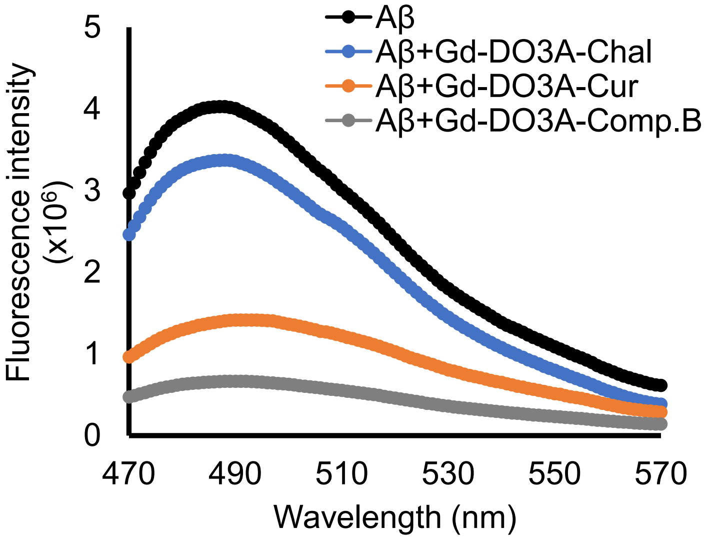

Figure S4. Inhibitory effect of the Gd probes toward Aβ aggregation measured by Thioflavin T assay

1. Experimental Procedure

All the solvents used were in analytical standard grade. The NMR spectra were measured on a Bruker biospin AVANCE II (400 MHz for ^{1}H and 100 MHz for ^{13}C) or a Bruker biospin AVANCE III (500 MHz for ^{1}H, 125 MHz for ^{13}C, and 470 MHz for ^{19}F). Chemical shift (δ) was reported in ppm relative to internal tetramethylsilane. The HRMS data were recorded on Bruker ESI-TOF-MS micrOTOF II instrument with sodium formate as the calibration standard. Vanilin, 3-bromopropylamine hydrobromide, potassium carbonate, potassium bicarbonate, morpholine, boric acid, and 10% Pd/C were purchased from Wako Chemical (Japan). The 1,4,7,10-tetraazacyclododecane were purchased from Accela (USA). Benzyl chloroformate, tert-butyl bromoacetate, PyBOP, and HOBt were purchased from Tokyo Chemical Industry (Japan). Microwave for synthesis was conducted on Biotage® Initiator+ instrument. Column chromatography was performed on silica gel Chromatorex (Japan). Purity analysis was determined by HPLC analysis using Inertsil ODS-3 5 µm (4.6 × 75 mm; GL Science) with a linear gradient of 0.1% formic acid in water/0.1% formic acid in MeCN detected by UV lamp for 20 min. Amyloid β (Aβ_{42}) peptide was purchased from Peptide Institute (Japan). Thioflavin T (ThT) for fluorescence detection of Aβ_{42} was purchased from Sigma (USA).

Synthesis of compound 2. Acetylacetone (10 mmol) and boric acid (10 mmol) were suspended in DMF. Compound 7 (2 mmol) was added then followed by morpholine (2 mmol). The mixture was irradiated in microwave at 100 °C for 10 minutes. The reaction mixture was quenched by HCl 0.1 N and extracted using ethyl acetate. The organic phase was dried using $MgSO_4$. The crude product was purified by column chromatography (silica gel, hexane:ethyl acetate 4:1) to obtain the desired compound.

(4-hydroxy-3-methoxyphenyl) hex-5-ene-2,4-dione (2). Yield: 65%. ^{1}H-NMR (400 MHz, $CDCl_3$): δ (ppm) 2.20 (-CH_3, s, 3H), 3.98 (-OCH_3, s, 3H), 5.68 (=CH, s, 1H), 5.87 (=CH, s, 1H), 6.37 (=CH, *J*=15.8, d, 1H), 6.97 (=CH, *J*=8.2, d, 1H), 7.06 (=CH, *J*=1.8 Hz, d, 1H), 7.13 (=CH, *J*=8.2, 1.8 Hz, dd, 1H), 7.58 (=CH, *J*=15.8 Hz, d, 1H). ^{13}C-NMR (125 MHz, $CDCl_3$): δ (ppm) 26.59, 55.86, 100.69, 111.39, 115.88, 119.87, 123.09, 126.55, 140.47, 148.19, 149.41, 178.48, 196.85. LRMS-ESI (*m/z*): calcd for $C_{13}H_{15}O_4^+$ $[M+H]^+$ 235.10; found 235.13.

Compound 2 was synthesized as described by Utomo et al.[1]

Synthesis of compound 3a or 3b. Vanillin or 4-hydroxy 3-trifluoromethyl benzaldehyde (6 mmol) and potassium carbonate (18 mmol) were dissolved in acetone 10 mL. The tert-butyl bromoacetate (7.5 mmol) was added then the mixture was refluxed for 2h. After removing of inorganic salt, the organic solvent was filtered and evaporated without further purification.

tert-butyl 2-(4-formyl-2-methoxyphenoxy)acetate (3a). Yield: 92%. ^{1}H-NMR (400 MHz, $CDCl_3$): δ (ppm) 1.42 (-CH_3, s, 9H), 3.88 (-OCH_3, s, 3H), 4.63 (-CH_2, s, 2H), 6.82 (=CH, *J*=8.6, d, 1H), 7.35-7.37 (m, 2H), 9.79 (-CHO, s, 1H). ^{13}C-NMR (100 MHz, $CDCl_3$): δ (ppm) 27.93, 55.98, 66.01, 82.66, 109.79, 112.03, 126.01, 130.84, 149.81, 152.62, 166.69, 190.72. HRMS-ESI (*m/z*): calcd for $C_{14}H_{17}O_5^-$ $[M-H]^-$ 265.1081; found 265.1145.

tert-butyl 2-(4-formyl-2-(trifluoromethyl)phenoxy)acetate (3b). Yield: 97%. ^{1}H-NMR (400 MHz, $CDCl_3$): δ (ppm) 1.48 (3x-CH_3, s, 9H), 4.75 (-CH_2, s, 2H), 7.02 (=CH, *J*=8.6 Hz, d, 1H), 8.05 (=CH, *J*=8.6, 2.0 Hz, dd, 1H), 8.16 (=CH, *J*=1.8 Hz, d, 1H), 9.95 (-CHO, s, 1H). ^{13}C-NMR (125 MHz, $CDCl_3$): δ (ppm) 27.46, 65.62, 82.69, 112.74, 119.34 (J_{C-F}=31.6 Hz, q), 122.71 (J_{C-F}=271.1 Hz, q), 128.61 (J_{C-F}=5.0 Hz, q), 134.79, 160.10, 166. 06, 189.36. HRMS-ESI (*m/z*): calcd for $C_{14}H_{15}F_3O_4Na^+$ $[M+Na]^+$ 327.0815; found 327.0827.

Compound 3b was synthesized as described by Utomo et al.[1]

Synthesis of compound 4a and 4b. Compound **2** (0.5 mmol) and boric acid (0.5 mmol) were suspended in DMF. Compound 3a or 3b (0.5 mmol) was added then followed by morpholine (0.5 mmol). The mixture was irradiated in microwave at 100 ºC for 10 minutes. The reaction mixture was quenched by HCl 0.1 N and extracted using ethyl acetate. The organic phase was dried using $MgSO_4$. The crude product was purified by column chromatography (silica gel, hexane:ethyl acetate 2:1) to obtain the desired compound.

tert-butyl 2-(4-((1E,6E)-7-(4-hydroxy-3-methoxyphenyl)-3,5-dioxohepta-1,6-dien-1-yl)-2-methoxyphenoxy)acetate (4a). Yield: 54%. ^{1}H-NMR (400 MHz, $CDCl_3$): δ (ppm) 1.48 (-CH_3, s, 9H), 3.93 (-OCH3, s, 6H), 4.62 (-CH_2, s, 2H), 5.8-6.0 (-CH_2, s, 2H), 6.48 (=CH, *J*=15.8 Hz, d, 1H), 6.49 (=CH, *J*=15.8 Hz, d, 1H), 6.78 (=CH, *J*=8.4 Hz, d, 1H), 6.92 (=CH, *J*=8.1, 1.0 Hz, dd, 1H), 7.01-7.12 (m, 4H), 7.57 (=CH, *J*=15.7Hz d, 1H), 7.59 (=CH, *J*=15.7 Hz, d, 1H). ^{13}C-NMR (125 MHz, DMSO-d_6): δ (ppm) 27.94, 55.91, 66.15, 82.44, 101.37, 109.98, 110.66, 113.09, 115.18, 121.39, 120.02, 122.39, 122.89, 127.24, 129.06, 139.92, 140.86, 147.25, 148.46, 149.14, 149.46, 167.55, 182.66, 183.79. LRMS-ESI (*m/z*): calcd for $C_{27}H_{30}O_8Na^+$ $[M+Na]^+$ 505.18; found 505.12.

tert-butyl 2-(4-((1E,6E)-7-(4-hydroxy-3-methoxyphenyl)-3,5-dioxohepta-1,6-dien-1-yl)-2-(trifluoromethyl)phenoxy)acetate (4b). Yield: 48%. ^{1}H-NMR (400 MHz, $CDCl_3$): δ (ppm) 1.51 (-CH_3, s, 9H), 3.99 (-OCH_3, s, 3H), 4.70 (-CH_2, s, 2H), 5.86 (-CH_2, S, 2H), 6.53 (=CH, *J*=16.0 Hz, d, 1H), 6.57 (=CH, *J*=16.0 Hz, d, 1H) 6.92 (=CH, *J*=8.6 Hz, d, 1H), 6.99 (=CH, *J*=8.0 Hz, d, 1H), 7.11 (=CH, s, 1H), 7.19 (=CH, *J*=7.9 Hz, d, 1H), 7.61-7.69 (=CH, m, 3H), 7.85 (=CH, s, 1H). ^{13}C-NMR (125 MHz, DMSO-d_6): δ (ppm) 28.09, 56.15, 65.89, 82.26, 101.86, 111.83, 114.46, 116.16, 118.19 (J_{C-F}=30.7 Hz, q), 121.54, 123.80 (J_{C-F}=271.1 Hz, q), 123.82, 124.37, 126.69, 127.38 (J_{C-F}=4.1 Hz, q), 128.24, 133.99, 138.31, 141.93, 148.47, 150.01, 157.06, 167.32, 182.04, 185.11. ^{19}F NMR (470 MHz, DMSO-d_6): δ 60.9. HRMS-ESI (*m/z*): calcd for $C_{27}H_{26}F_3O_7^-$ $[M-H]^-$ 519.1636; found 519.1702.

Compound 4b was synthesized as described by Utomo et al.[1]

Synthesis of compound 5a and 5b. Compound **4a** or **4b** (0.41 mmol) was dissolved in dichloromethane 10 mL. Trifluoroacetic acid (8 mL, excess) was added then stirred for 2 h under argon gas in RT. The solvent was removed by evaporation and the product was purified by preparative HPLC (MeCN/water).

2-(4-((1E,6E)-7-(4-hydroxy-3-methoxyphenyl)-3,5-dioxohepta-1,6-dien-1-yl)-2-methoxyphenoxy)acetic acid (5a). Yield: 63%. ^{1}H-NMR (400 MHz, CD_3CN): δ (ppm) 3.92 (-CH_3, s, 3H), 3.94 (-OCH_3, s, 3H), 4.73 (-CH_2, s, 2H), 5.96 (-CH_2, s, 2H), 6.71 (=CH, *J*=15.7 Hz, d, 1H), 6.75 (=CH, *J*=15.7 Hz, d, 1H) 6.89 (=CH, *J*=8.1 Hz, d, 1H), 6.91 (=CH, *J*=8.1 Hz, d, 1H), 7.17 (=CH, *J*=8.3 Hz, d, 1H), 7.19 (=CH, *J*=8.3 Hz, d, 1H), 7.29 (=CH, *J*=8.2 Hz, d, 1H), 7.61 (=CH, *J*=15.8 Hz, d, 1H), 7.62 (=CH, *J*=15.8 Hz, d, 1H). ^{13}C-NMR (125 MHz, DMSO-d_6): δ (ppm) 49.07, 56.08, 65.54, 101.51, 111.38, 113.25, 116.19, 121.55, 122.73, 122.89, 123.61, 126.77, 128.59, 140.47, 141.45, 148.46, 149.51, 149.81, 149.91, 162.79, 170.54, 183.06, 184.25. HRMS-ESI (m/z): calcd for $C_{23}H_{21}O_8^-$ $[M-H]^-$ 425.1242; found 425.1215

2-(4-((1E,6E)-7-(4-hydroxy-3-methoxyphenyl)-3,5-dioxohepta-1,6-dien-1-yl)-2-(trifluoromethyl)phenoxy)acetic acid (5b). Yield: 56%. ^{1}H-NMR (400 MHz, CD_3CN): δ (ppm) 3.98 (-OCH_3, s, 3H), 4.84 (-CH_2, s, 2H), 5.98 (=CH, s, 1H), 6.72 (=CH, *J*=16.0 Hz, d, 1H), 6.78 (=CH, *J*=16.0 Hz, d, 1H), 6.89 (=CH, *J*=8.0 Hz, d, 1H), 7.10 (=CH, *J*=8.8 Hz, d, 1H), 7.18 (=CH, *J*=8.2, 1.9 Hz, dd, 1H), 7.29 (=CH, *J*=1.8 Hz, d, 1H), 7.65 (=CH, *J*=15.6 Hz, d, 2H), 7.84 (=CH, *J*=8.6 Hz, d, 1H), 7.94 (=CH, *J*=1.9 Hz, d, 1H). ^{13}C-NMR (125 MHz, DMSO-d_6): δ (ppm) 55.92, 66.81, 101.58, 111.59, 114.52, 115.99, 117.66 (J_{C-F}=30.2 Hz, q), 121.29, 122.67, 123.58, 123.74 (J_{C-F}=270.7 Hz, q), 126.40, 126.62, 126.74, 127.04 (J_{C-}

$_F$=4.7 Hz, q), 127.22, 127.49 (J_{C-F}=5.2 Hz, q), 133.61, 138.37, 141.62, 141.98, 148.28, 157.84, 170.25, 182.01, 184.68, 198.26. ^{19}F NMR (470 MHz, DMSO-d$_6$): δ 60.8. HRMS-ESI (*m/z*): calcd for $C_{23}H_{18}F_3O_7^-$ [M-H]$^-$ 463.1010; found 463.1087.

Synthesis of compound 6. Synthesis of compound **6** followed the reported method with slight modification[2]. The 3-bromopropylamine hydrobromide (2.05 g, 10 mmol) and NaOH 2M (10 mL, 20 mmol) was dissolved in cold dioxane, then benzyl chloroformate (1.43 mL, 10 mmol) was added dropwise. The reaction was mixed and stirred at room temperature. After overnight stirring, dioxane was removed by evaporation, then the remaining residue was extracted by ethyl acetate. The organic layer was washed by $NaHCO_3$ aq., water, brine, then was dried by Na_2SO_4, and evaporated to obtain crude product for next steps without further purification. The crude product (0.5 g, 2.1 mmol) was added dropwise in the 1,4,7,10-tetraazacyclododecane (0.5 g, 2.9 mmol) dissolved in toluene, then refluxed overnight under argon gas. The mixture was extracted with water then separated by dichloromethane, washed with brine, dried with Na_2SO_4, and evaporated to obtain crude product for next steps without further purification. The crude product (0.5 g, 1.4 mmol) and $KHCO_3$ (1.1g, 14 mmol) was dissolved in acetonitrile. Tert-butyl bromoacetate (0.81g, 4.2 mmol) was added dropwise and refluxed overnight under argon gas. The $KHCO_3$ was filtered, and the filtrate was evaporated. The crude product (0.2 g, 0.1 mmol) and Pd/C 10% (20 mg) was dissolved in ethanol then stirred overnight under hydrogen gas. After filtration, the filtrate was evaporated to get white solid powder product.

Benzyl (3-(1,4,7,10-tetraazacyclododecan-1-yl)propyl)carbamate (6). Yield: 61%. ^{1}H-NMR (400 MHz, CD_3CN): δ (ppm) 1.44 (-CH_3, s, 27H), 1.72 (-CH_2, broad s, 2H), 2.51-3.51 (m, 24H), 3.92 (-NH_2, broad s, 2H). ^{13}C-NMR (100 MHz, CD_3CN): δ (ppm) 24.21, 28.09, 39.48, 50.58, 50.81, 51.31, 53.23, 57.52, 57.79, 82.22, 82.61, 171.76, 173.21. HRMS-ESI (*m/z*): calcd for $C_{29}H_{58}N_5O_6^+$ [M+H]$^+$ 572.4382; found 572.4355.

Synthesis of compound 7a and 7b. Compound **5a** or **5b** (0.18 mmol), HOBt (0.18 mmol) and PyBOP (0.18 mmol) were dissolved in DMF (3 mL). Compound **6** (0.22 mmol) and triethylamine (0.54 mmol) in DMF 2 mL were added followed by stirring for 24 h. The desired product was purified by column chromatography (silica gel, dichloromethane/methanol).

tri-tert-butyl 2,2',2''-(10-(3-(2-(4-((1E,6E)-7-(4-hydroxy-3-methoxyphenyl)-3,5-dioxohepta-1,6-dien-1-yl)-2-methoxyphenoxy)acetamido)propyl)-1,4,7,10-tetraazacyclododecane-1,4,7-triyl)triacetate (7a). Yield 64%. ^{1}H-NMR (400 MHz, CD_3CN): δ (ppm) 1.46 (-CH_3, s, 27H), 2.70-3.80 (m, 24h), 3.93 (-OCH_3, s, 6h), 4.55 (-CH_2, s, 1H), 4.78 (-CH_2, s, 1H), 5.96 (-CH_2, s, 1H), 6.69 (=CH, *J*=15.7 Hz, d, 1H), 6.75 (=CH, *J*=15.7 Hz, d, 1H), 6.89 (=CH, *J*=8.1 Hz, d, 1H), 6.98 (=CH, *J*=8.2 Hz, d, 1H), 7.16 (=CH, *J*=8.1 Hz, d, 1H), 7.21 (=CH, *J*=8.8 Hz, d, 1H), 7.27-7.32 (m, 2H), 7.60 (=CH, *J*=15.7 Hz, d, 1H), 7.61 (=CH, *J*=15.7 Hz, d, 1H). ^{13}C-NMR (125 MHz, DMSO-d$_6$): δ (ppm) 28.24, 28.27, 46.97, 49.26, 51.99, 52.30, 52.53, 55.91, 56.13, 68.57, 81.15, 101.50, 111.41, 111.83, 113.59, 114.44, 116.18, 121.53, 122.87, 122.97, 123.10, 123.67, 126.71, 129.34, 140.21, 141.59, 148.46, 149.57, 149.67, 149.86, 149.95, 168.19, 169.44, 170.52, 170.71, 182.82, 184.48. HRMS-ESI (m/z): calcd for $C_{52}H_{78}N_5O_{13}^+$ [M+H]$^+$ 980.5591; found 980.5518.

tri-tert-butyl 2,2',2''-(10-(3-(2-(4-((1E,6E)-7-(4-hydroxy-3-methoxyphenyl)-3,5-dioxohepta-1,6-dien-1-yl)-2-(trifluoromethyl)phenoxy)acetamido)propyl)-1,4,7,10-tetraazacyclododecane-1,4,7-triyl)triacetate (7b). Yield 57%. ^{1}H-NMR (500 MHz, DMSO-d$_6$): δ (ppm) 1.41 (-CH_3, s, 27H), 2.6-3.8 (m, 24h), 3.84 (-OCH_3, s, 3h), 4.73 (-CH_2, s, 2H), 6.1 (-CH_2, s, 1H), 6.77 (=CH, *J*=15.7 Hz, d, 1H), 6.84 (=CH, *J*=8.1, d, 1H), 6.91 (=CH, *J*=15.6, d, 1H), 7.13 (=CH, *J*=8.4, d, 1H), 7.33 (=CH, s, 1H), 7.58 (=CH, *J*=15.7

Hz, d, 1H), 7.62 (=CH, *J*=15.3 Hz, d, 1H), 7.93 (=CH, *J*=8.6 Hz, d, 1H), 7.97 (=CH, s, 1H), 8.06 (=CH, *J*=8.1 Hz, d, 1H). ^{13}C-NMR (125 MHz, DMSO-d$_6$): δ (ppm) 27.90, 28.22, 30.34, 52.46, 56.14, 56.26, 65.56, 66.56, 68.98, 70.27, 81.11, 101.79, 111.83, 114.66, 116.20, 117.95 ($J_{C\text{-}F}$=30.5 Hz, q), 121.51,123.79, 123.93 ($J_{C\text{-}F}$=270.7 Hz, q), 124.01, 126.62, 127.29 ($J_{C\text{-}F}$=5.2 Hz, q), 127.64, 128.32, 133.88, 138.51, 141.86, 148.49, 150.12, 157.82, 170.10, 182.18, 184.98. ^{19}F NMR (470 MHz, DMSO-d$_6$): δ 60.8. HRMS-ESI (m/z): calcd for $C_{52}H_{75}F_3N_5O_{12}^+$ $[M+H]^+$ 1018.5359; found 1018.5384.

Synthesis of compound 8a and 8b. Compound **7a** or **7b** (0.18 mmol) was dissolved in dichloromethane 10 mL. Trifluoroacetic acid (8 mL, excess) was added then stirred for 2 h under argon gas in RT. The solvent was removed by evaporation and the product was purified by preparative HPLC (MeCN/water).

2,2',2''-(10-(3-(2-(4-((1E,6E)-7-(4-hydroxy-3-methoxyphenyl)-3,5-dioxohepta-1,6-dien-1-yl)-2-methoxyphenoxy)acetamido)propyl)-1,4,7,10-tetraazacyclododecane-1,4,7-triyl)triacetic acid (8a). Yield 39%. ^{1}H-NMR (400 MHz, CD$_3$CN): δ (ppm) 3.3-4.0 (m, 24H), 3.93 (-OCH$_3$, s, 6H), 4.61 (-CH$_2$, s, 2H), 5.99 (=CH, s, 1H), 6.69 (=CH, *J*=15.8 Hz, d, 1H), 6.76 (=CH, *J*=15.9 Hz, d, 1H), 6.91 (=CH, *J*=8.1 Hz, d, 1H), 6.99 (=CH, *J*=8.8 Hz, d, 1H), 7.27 (=CH, s, 1H), 7.33 (=CH, s, 1H), 7.62 (=CH, *J*=15.8 Hz, d, 2H), 8.04 (=CH, s, 1H). LRMS-ESI (m/z): calcd for $C_{40}H_{52}N_5O_{13}^-$ $[M-H]^-$ 810.36; found 810.76.

2,2',2''-(10-(3-(2-(4-((1E,6E)-7-(4-hydroxy-3-methoxyphenyl)-3,5-dioxohepta-1,6-dien-1-yl)-2-(trifluoromethyl)phenoxy)acetamido)propyl)-1,4,7,10-tetraazacyclododecane-1,4,7-triyl)triacetic acid (8b). Yield 34%. ^{1}H-NMR (500 MHz, DMSO-d$_6$): δ (ppm) 2.6-3.7 (m, 24H), 3.84 (-OCH$_3$, s, 3H), 4.65 (-CH$_2$, s, 2H) 6.77 (=CH, *J*=15.6 Hz, d, 1H), 6.88 (=CH, *J*=15.7 Hz, d, 1H), 7.09 (=CH, *J*=7.8 Hz, d, 1H), 7.15 (=CH, *J*=7.7 Hz, d, 1H), 7.2-7.4 (m, 2H) 7.57 (=CH, *J*=15.4 Hz, d, 1H), 7.61 (=CH, *J*=15.4 Hz, d, 1H), 7.79 (=CH, *J*=7.9, 2.7 Hz, dd, 1H), 7.91 (=CH, *J*=7.7 Hz, d, 1H), 7.95 (=CH, s, 1H). ^{19}F NMR (470 MHz, DMSO-d$_6$): δ 60.81. LRMS-ESI (m/z): calcd for $C_{40}H_{49}F_3N_5O_{12}^-$ $[M-H]^-$ 848.3335; found 848.3722.

Preparation of Gd-DO3A-Cur and Gd-DO3A-Comp.B. Compound **8a** and **8b** were mixed with equimolar quantities of $GdCl_3$ in Milli-Q water. The pH of the solution was adjusted to **7** by adding NaOH 2 mM. The solution was allowed to react for 1 hours at 60 °C by regularly controlling the pH. The absence of free metal was checked in each sample by using the xylenol orange test and purified by preparative HPLC (MeCN/water).

Gd-DO3A-Cur. LRMS-ESI (m/z): calcd for $C_{40}H_{49}GdN_5O_{13}^-$ $[M-H]^-$ 965.26; found 965.32.

Gd-DO4A-Comp.B. LRMS-ESI (m/z): calcd for $C_{40}H_{47}F_3GdN_5O_{12}Cl^-$ $[M+Cl]^-$ 1039.21; found 1038.82.

Aβ Preparation for Aggregation Study

To prepare the Aβ monomer stock, in amount 0.5 mg of the lyophilized Aβ$_{42}$ (Peptide Institute) was dissolved in DMSO by gently mixing without vortexing to obtain 500 µM as final concentration. The solution was centrifuged at 13,200 rpm at 4 °C for 10 minutes. The supernatant was collected and stored in −80 °C until used. The Aβ stock was diluted into 100 µM in 2 mM NaOH freshly before used.

Congo Red and Thioflavin T Assay

Congo Red or Thioflavin T stock at concentration 2 mM was freshly prepared in tris glycine 10 mM pH 8.5, then diluted in PBS at pH 7.4 to reach final concentration 40 µM. Gd probes was added at final concentration 10 µM followed by Aβ to reach final concentration 20 µM then was incubated at 37 °C. After 24 h, the mixture was transferred in 384-well plate black bottom non-binding to be scanned the fluorescence

intensity with an excitation of 430 nm and emission range from 450 to 600 nm using microplate reader (SpectraMax iD5, Molecular Device, USA).

Negative-Staining TEM Imaging

Elastic carbon grids (ELS-C10, STEM, Japan) was hydrolysed by ion coater (IB-2, Eiko, Japan) with 3 mA of plasma current for 40 seconds before applying sample solution. Briefly, 5 μL of the Aβ only or containing Gd probes after 24 h incubation was applied to a hydrophilic grid and incubated for one minute at RT. After gently dried with filter paper, the grid sample was washed with Milli-Q water and dried again with filter paper two times. Finally, the grid was incubated with 5 μL of 1% Nano-W negative staining solution (NY, USA) for one minute followed by complete drying using filter paper. The negative stained sample was observed using TEM H-8100 (Hitachi) operated at 200 kV.

Nuclear Magnetic Resonance (NMR) Relaxometry

Longitudinal relaxation time (T_1) was measured using a Spinsolve ULTRA 43 MHz ^{1}H-NMR (Magritek Ltd., Wellington, New Zealand) in PBS at pH 7.4. The inversion-recovery (IR) was used to measure T_1. The parameters in the IR pulse sequence were as follows: number of scans = 2, acquisition time = 1.6 s, repetition time = 7 s, maximum inversion time = 5 s, number of steps = 21. To confirm inhibitory effect on fibril growth, 20 μM of monomeric Aβ was mixed with 10 μM Gd probes at 37 °C and the T_1 was measured at 0.5, 1, 2, and 24 h after preparing the samples. The fibrillation growth was detected as follows: 20 μM of monomeric Aβ was pre-incubated at 37 °C for 1, 3, 6, 12, and 24 h to cause the fibril formation in different growth stages. Then the 10 μM Gd probes were added into the fibril at each incubation time and T_1 was measured without further incubation. The T_1 change (ΔT_1) was calculated by the equation as follow;

$$\Delta T_1 = \frac{(T_{1,(t)} - T_{1,(0)})}{T_{1,(0)}} \times 100\ (\%)$$

where $T_{1,(t)}$ is T_1 of the Gd probe solution with Aβ at *t* hour while $T_{1,(0)}$ was T_1 of the Gd probe solution without Aβ at 0 hour.

***In Vitro* MRI Imaging**

MRI was performed with a benchtop 1 tesla MRI scanner (ICON, Bruker BioSpin, Ettlingen, Germany). A 100 μL of each sample was added into 0.2 mL PCR tubes and imaged using a solenoid volume coil for transmission and reception. An inversion recovery rapid acquisition with relaxation enhancement (IR-RARE) sequence was used to obtain multi-inversion time images, with parameters including matrix size = 100 × 50, field of view = 4.0 cm × 2.0 cm, slice thickness = 3 mm, repetition time = 10,000 ms, effective echo time (TE) = 10 ms, RARE factor = 4, the number of excitations = 1, inversion time = 100, 300, 500, 700, 900, 1100, 1300, 1500, 1700, 1900, 2100, 2300, 2500, 2700, 2900, 3100, 3300, 3500, 3700, 3900 ms.

Cell Culture

The Neuro 2a cells were suspended in Dulbecco's Modified Eagle Medium (DMEM) medium supplemented with 10% fetal bovine serum. Briefly, Nl.2A cells ($5x10^3$ cells/well) was grown on 96-well plate overnight at 37 °C CO_2 5%. For the cytotoxicity study, cells were treated with Gd compounds at serial concentration (10, 25, 50, 100, 200, 500 μM) for 24 hours.

MTT Assay

An MTT reduction assay was conducted as described previously[3]. Briefly, MTT powder was dissolved in PBS pH 7.4 to obtain 5 mg/mL concentration stock, then was diluted into 0.5 mg/mL in DMEM medium. After removal of medium on 96-well plate containing-treated cell, each well was added by 100 µL MTT 0.5 mg/mL and incubated at 37 ºC CO_2 5%. After 3 h incubation, 100 µL DMSO was added following the absorbance measurement at wavelength of 550 nm using microplate reader (Tecan Infinite F200, Tecan, Switzerland). Calculation of % cells viability was measured by dividing the absorbance of untreated cells with the absorbance of curcumin-treated cells.

References

1 R. Y. Utomo, Y. Asawa, S. Okada, H. S. Ban, A. Yoshimori, J. Bajorath and H. Nakamura, *Bioorg. Med. Chem.*, 2021, **46**, 116357.

2 E. Pazos, M. Goličnik, J. L. Mascareñas and M. E. Vázquez, *Chem. Commun.*, 2012, **48**, 9534–9536.

3 T. Mosmann, *J. Immunol. Methods*, 1983, **65**, 55–63.

^{1}H-NMR Profile of tri-tert-butyl 2,2',2''-(10-(3-aminopropyl)-1,4,7,10-tetraazacyclododecane-1,4,7-triyl)triacetate

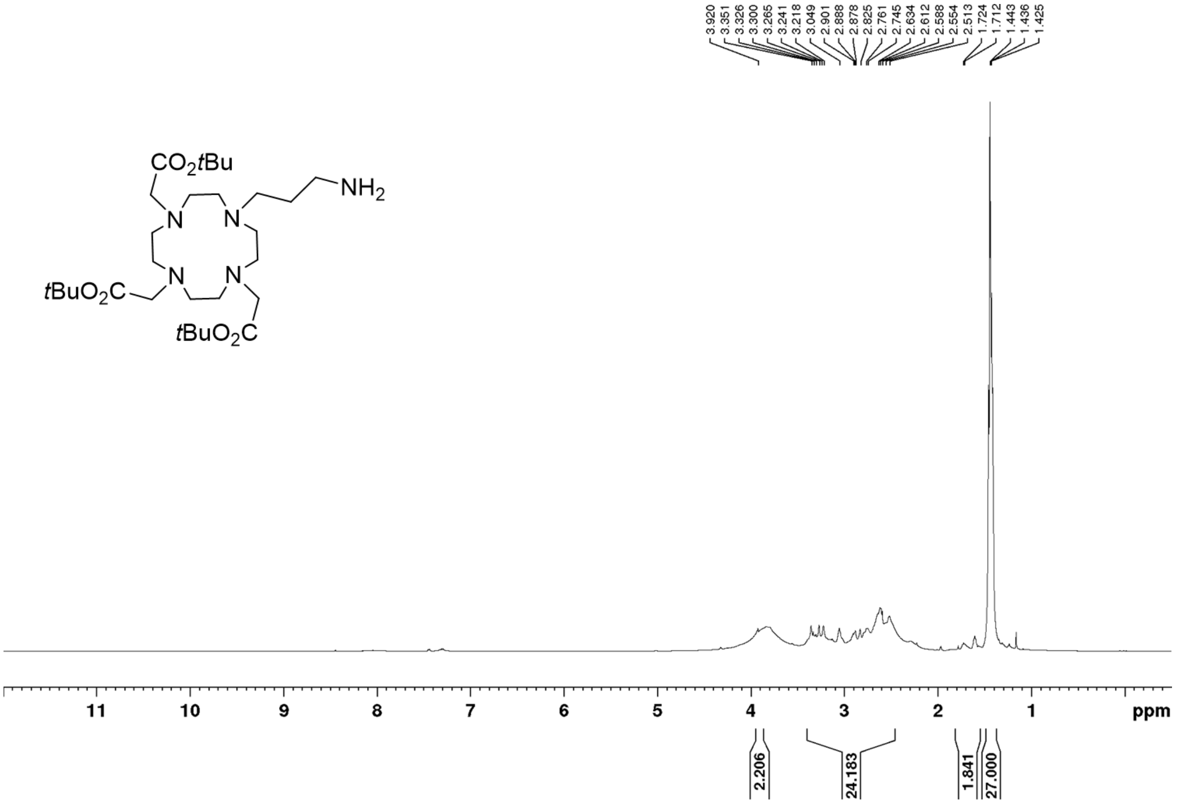

^{13}C-NMR Profile of tri-tert-butyl 2,2',2''-(10-(3-aminopropyl)-1,4,7,10-tetraazacyclododecane-1,4,7-triyl)triacetate

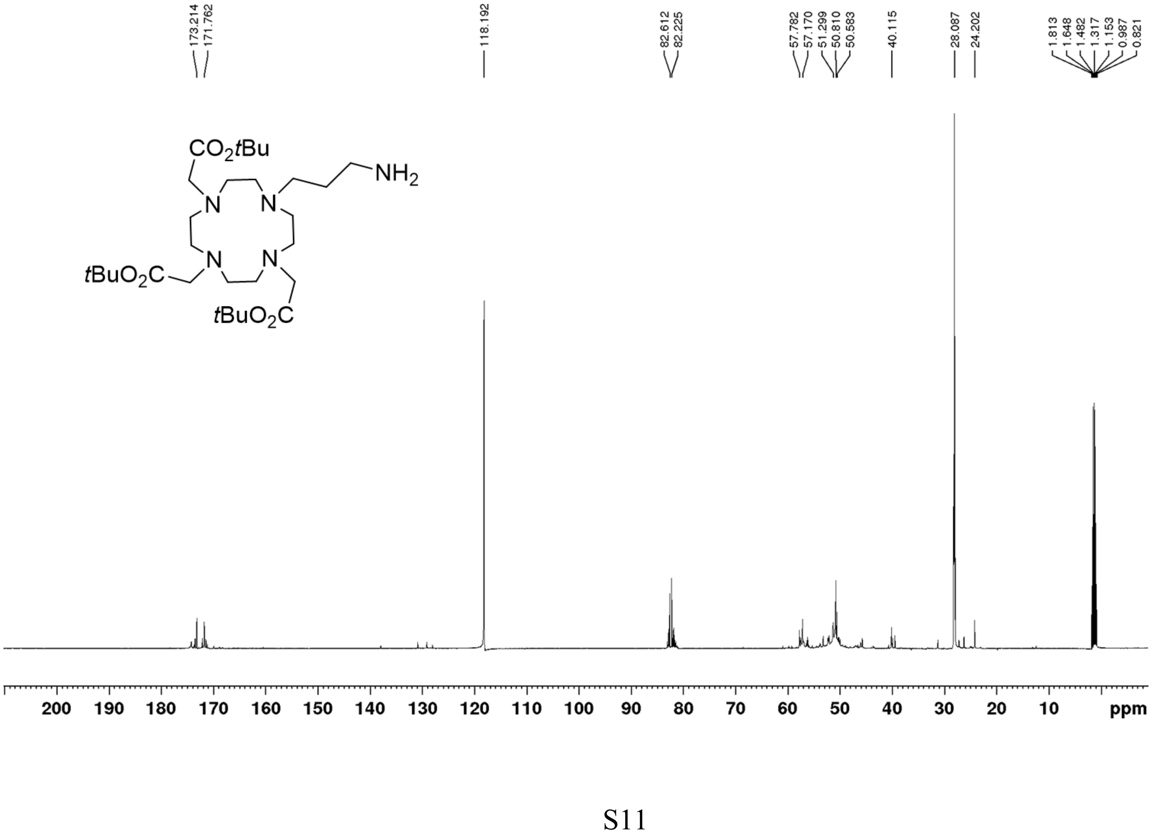

^{1}H-NMR Profile of tert-butyl 2-(4-formyl-2-methoxyphenoxy)acetate

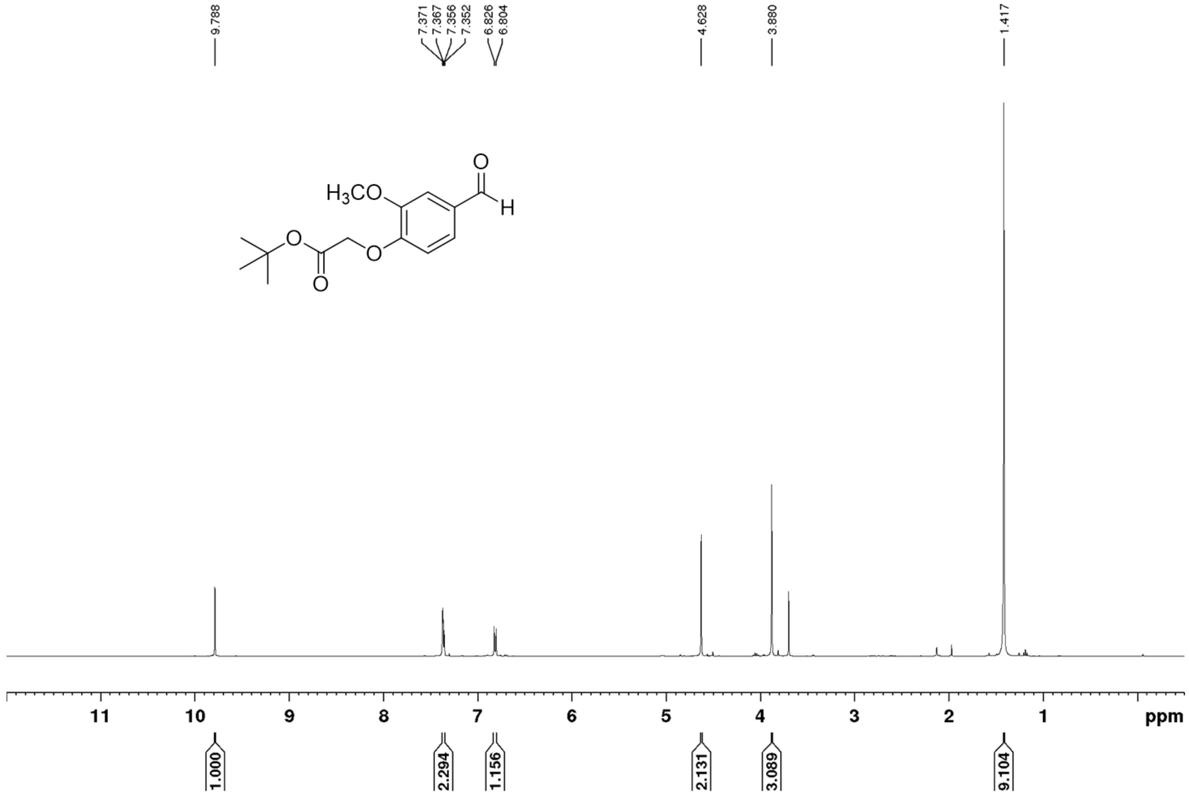

^{13}C-NMR Profile of tert-butyl 2-(4-formyl-2-methoxyphenoxy)acetate

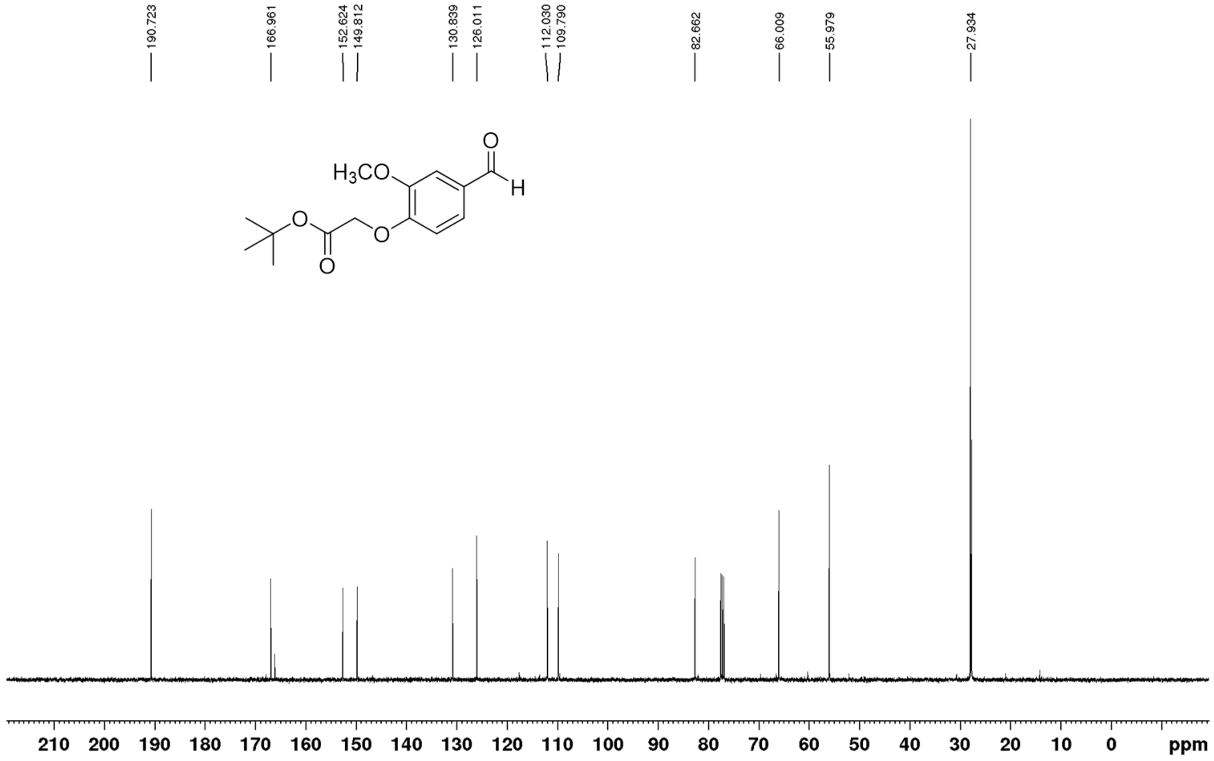

^{1}H-NMR Profile of methyl 2-(4-formyl-2-(trifluoromethyl)phenoxy)acetate

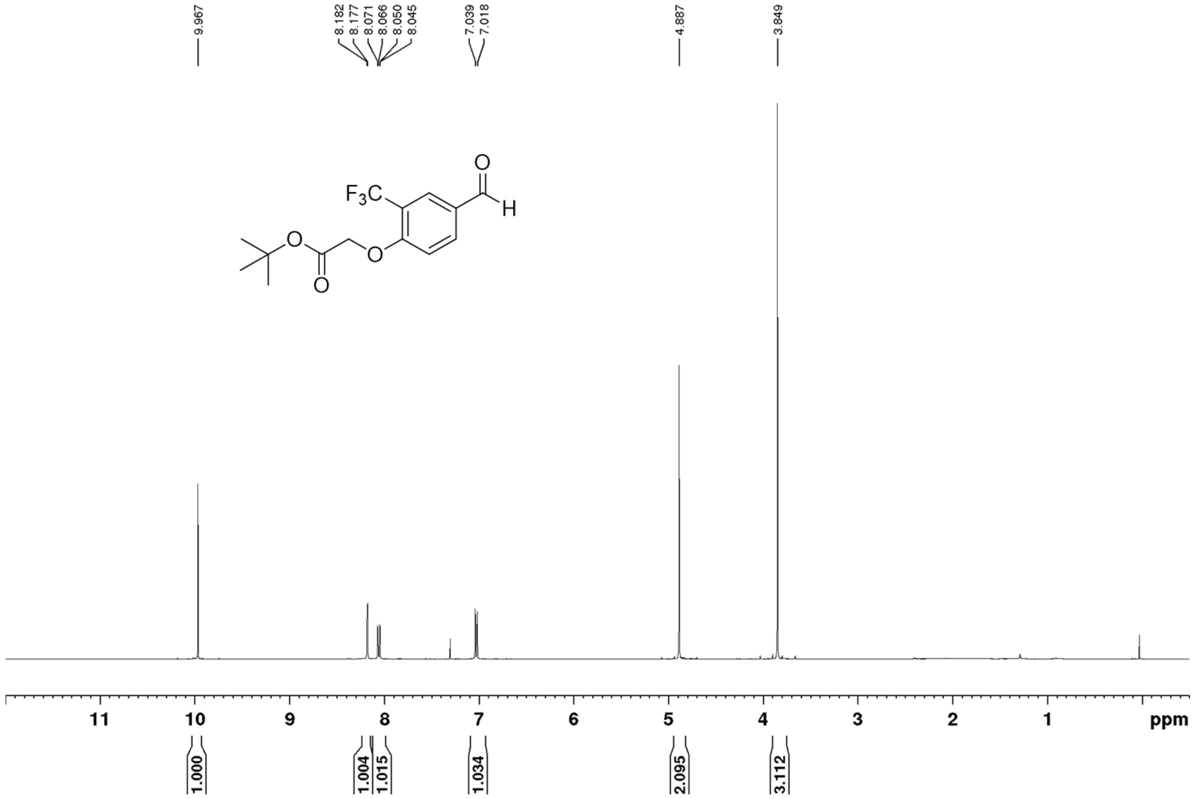

^{13}C-NMR Profile of methyl 2-(4-formyl-2-(trifluoromethyl)phenoxy)acetate

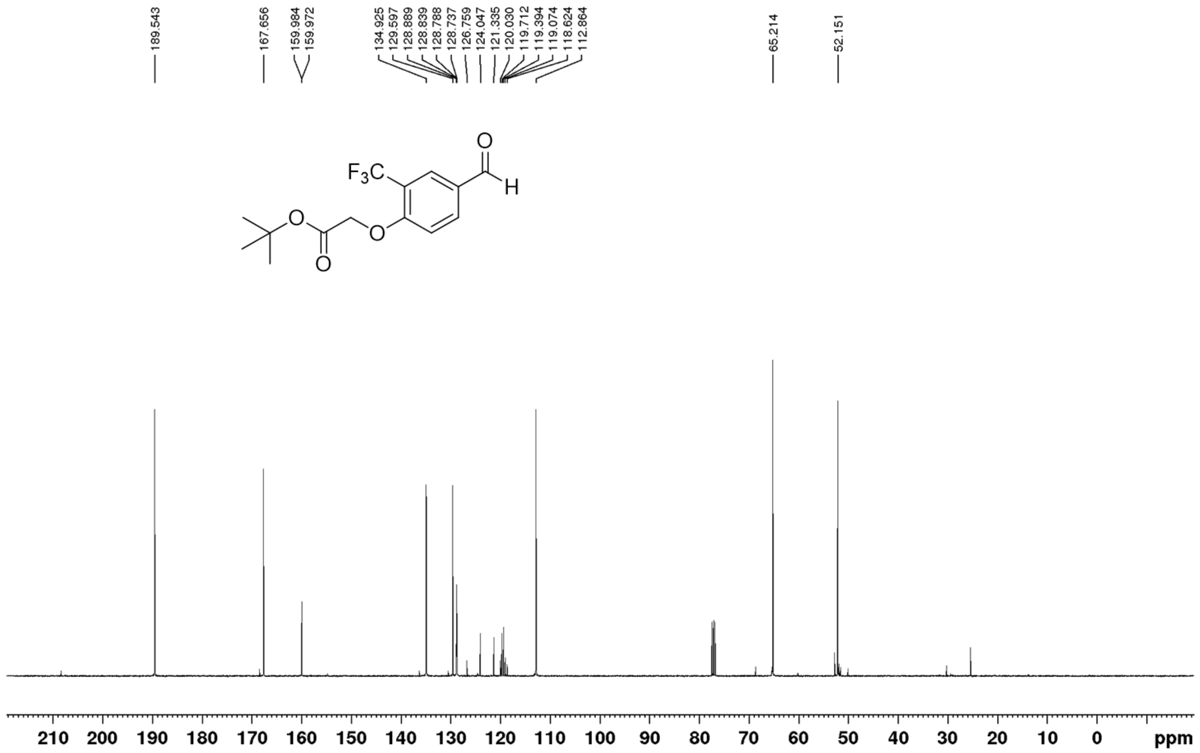

^{1}H-NMR Profile of (E)-6-(4-hydroxy-3-methoxyphenyl)hex-5-ene-2,4-dione

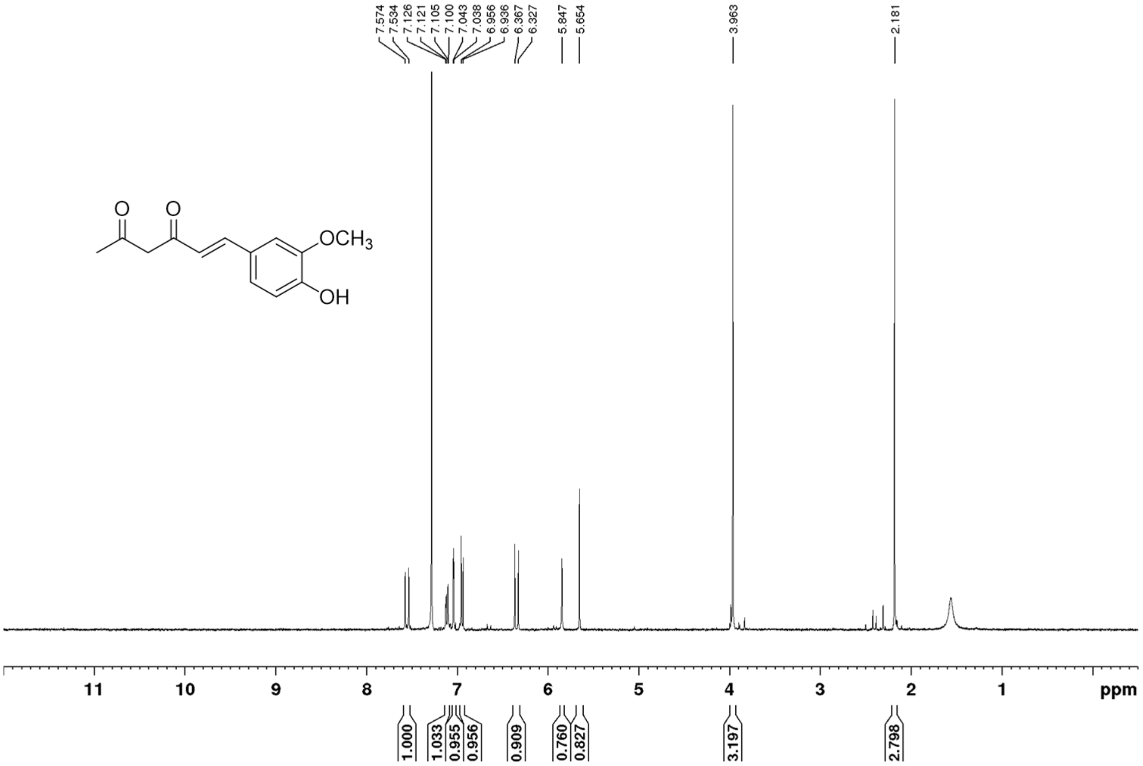

^{13}C-NMR Profile of (E)-6-(4-hydroxy-3-methoxyphenyl)hex-5-ene-2,4-dione

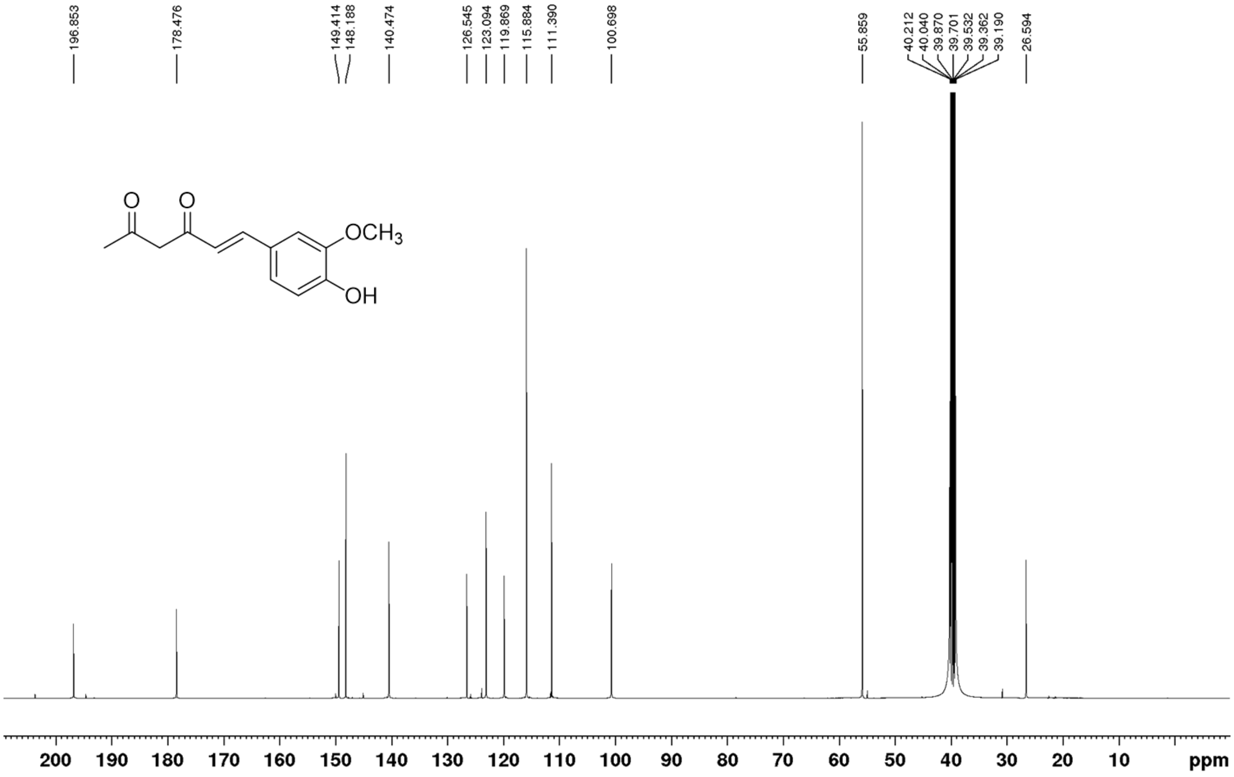

^{1}H-NMR Profile of tert-butyl 2-(4-((1E,6E)-7-(4-hydroxy-3-methoxyphenyl)-3,5-dioxohepta-1,6-dien-1-yl)-2-methoxyphenoxy)acetate

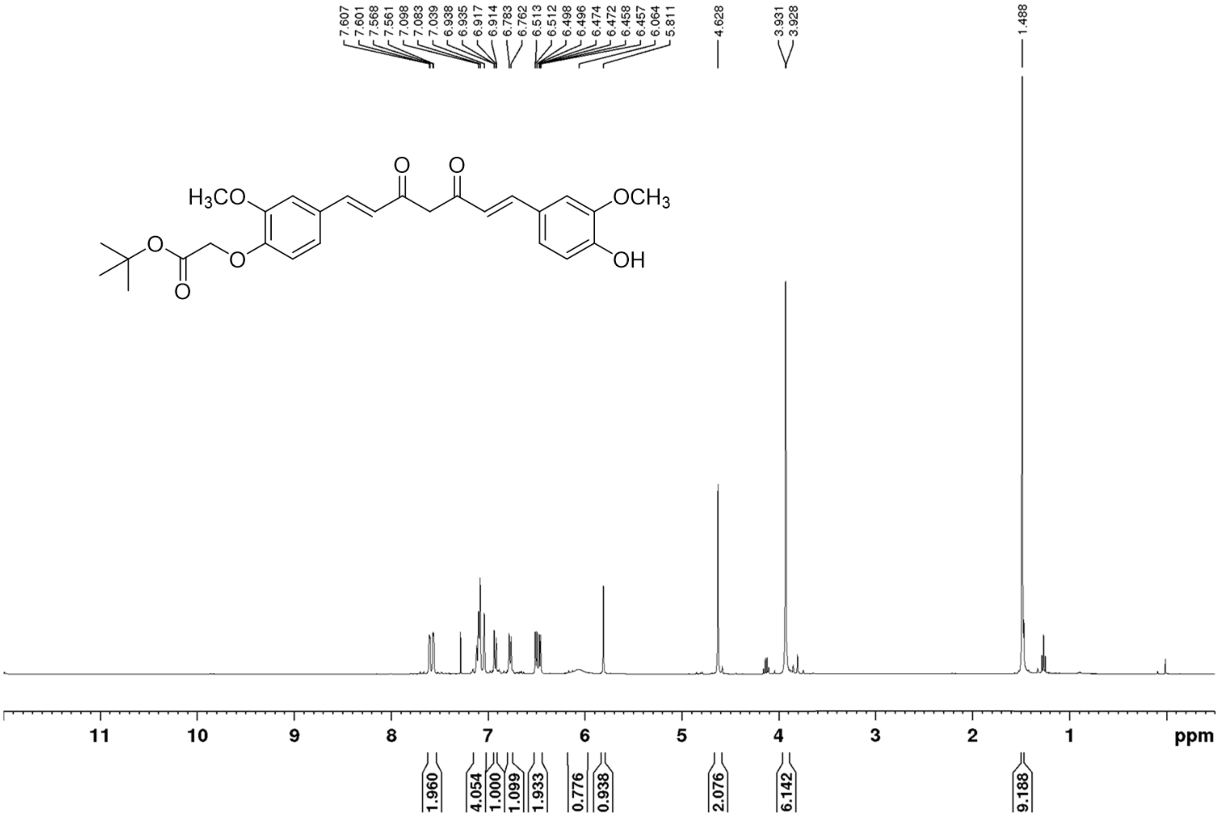

^{13}C-NMR Profile of tert-butyl 2-(4-((1E,6E)-7-(4-hydroxy-3-methoxyphenyl)-3,5-dioxohepta-1,6-dien-1-yl)-2-methoxyphenoxy)acetate

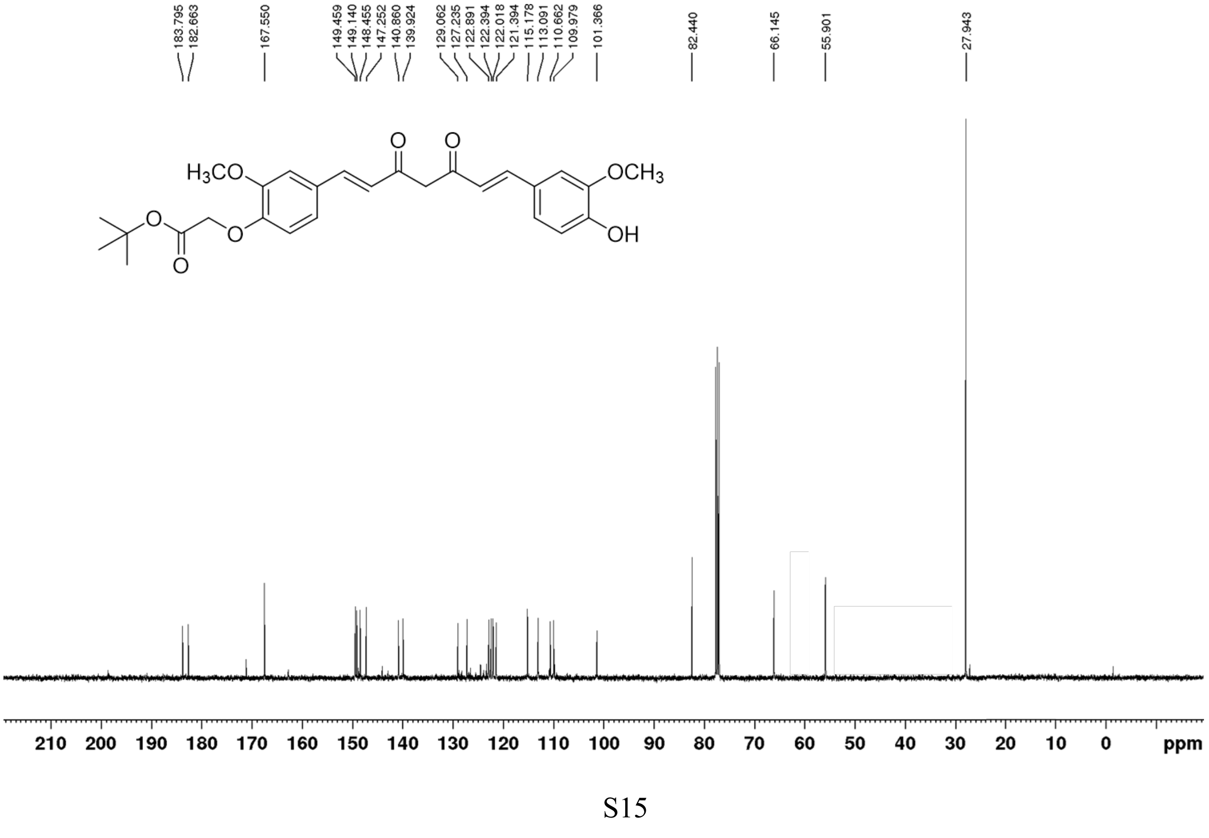

^{1}H-NMR Profile of tert-butyl 2-(4-((1E,6E)-7-(4-hydroxy-3-methoxyphenyl)-3,5-dioxohepta-1,6-dien-1-yl)-2-(trifluoromethyl)phenoxy)acetate

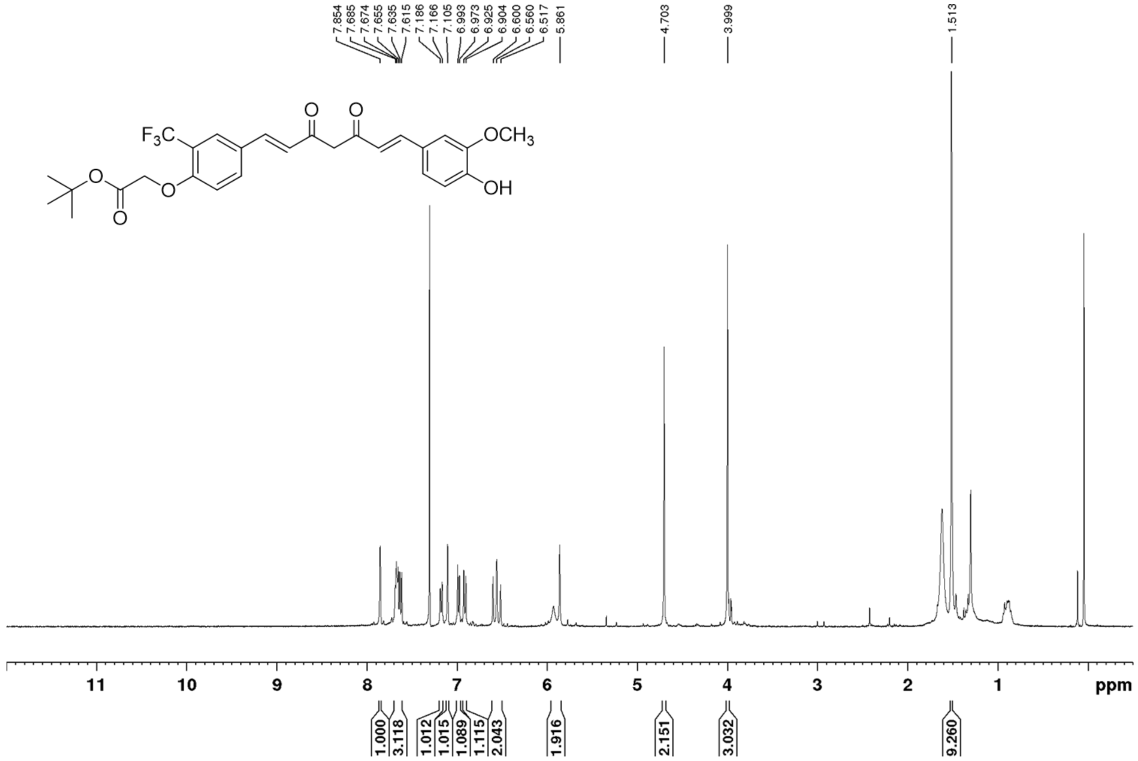

^{13}C-NMR Profile of tert-butyl 2-(4-((1E,6E)-7-(4-hydroxy-3-methoxyphenyl)-3,5-dioxohepta-1,6-dien-1-yl)-2-(trifluoromethyl)phenoxy)acetate

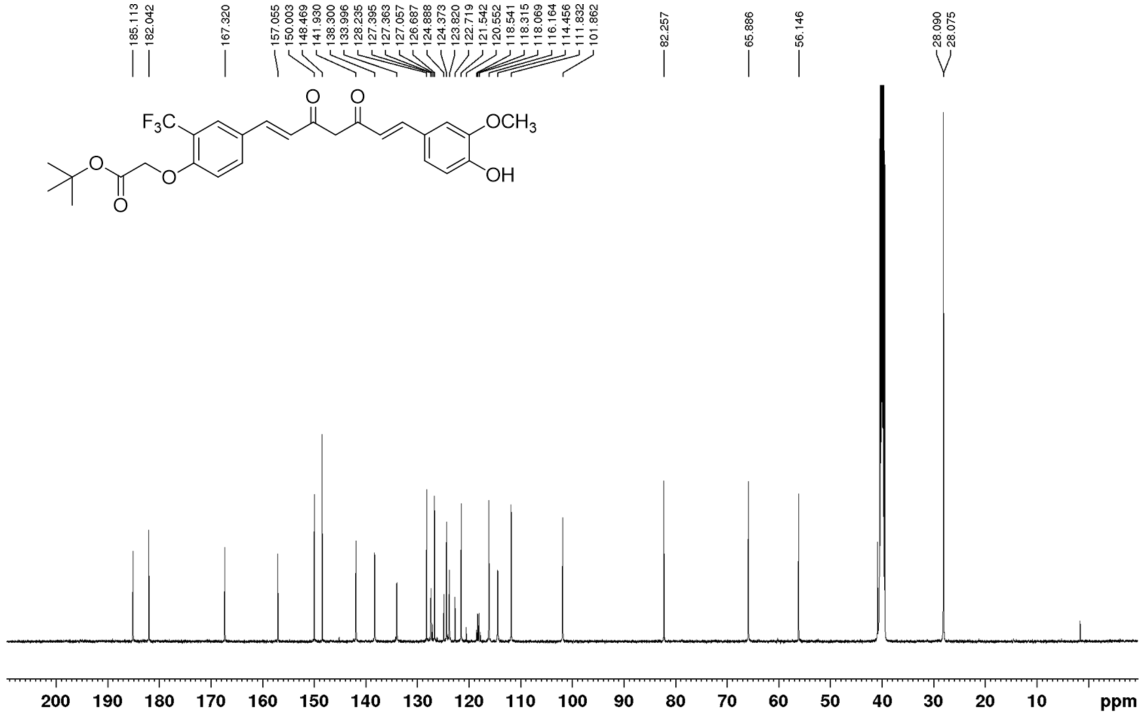

^{19}F-NMR Profile of tert-butyl 2-(4-((1E,6E)-7-(4-hydroxy-3-methoxyphenyl)-3,5-dioxohepta-1,6-dien-1-yl)-2-(trifluoromethyl)phenoxy)acetate

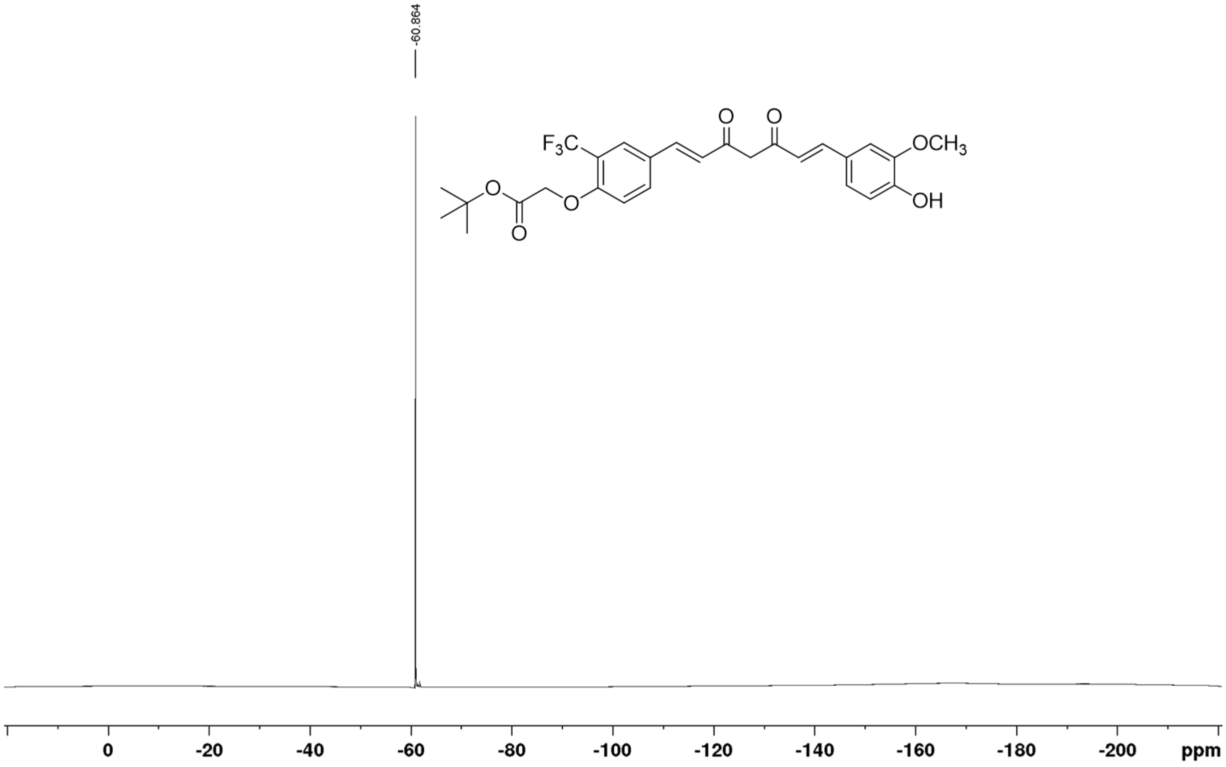

^{1}H-NMR Profile of 2-(4-((1E,6E)-7-(4-hydroxy-3-methoxyphenyl)-3,5-dioxohepta-1,6-dien-1-yl)-2-methoxyphenoxy)acetic acid

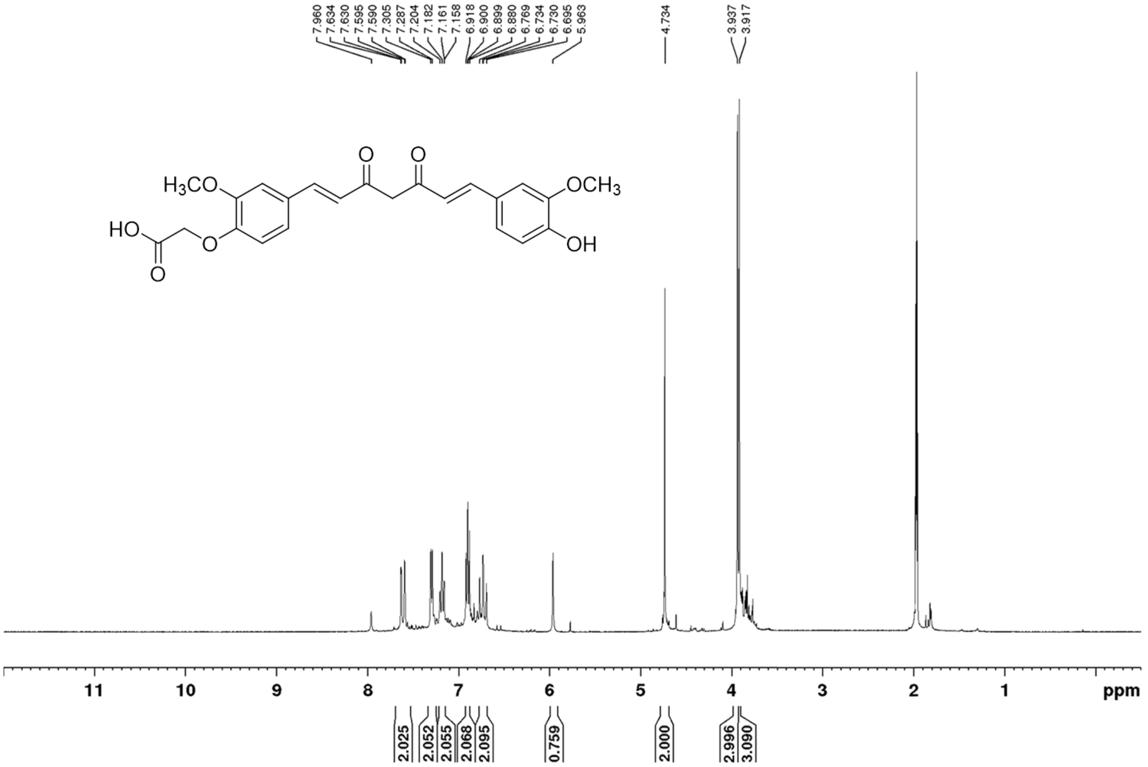

^{13}C-NMR Profile of 2-(4-((1E,6E)-7-(4-hydroxy-3-methoxyphenyl)-3,5-dioxohepta-1,6-dien-1-yl)-2-methoxyphenoxy)acetic acid

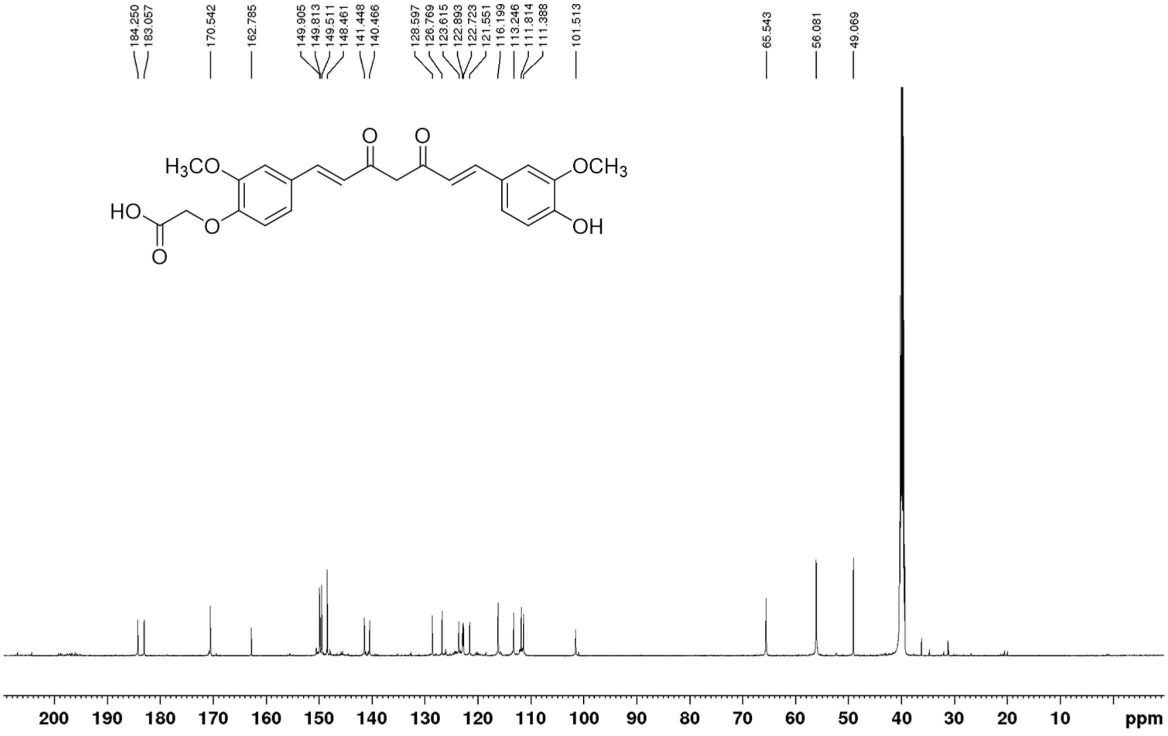

^{1}H-NMR Profile of 2-(4-((1E,6E)-7-(4-hydroxy-3-methoxyphenyl)-3,5-dioxohepta-1,6-dien-1-yl)-2-(trifluoromethyl)phenoxy)acetic acid

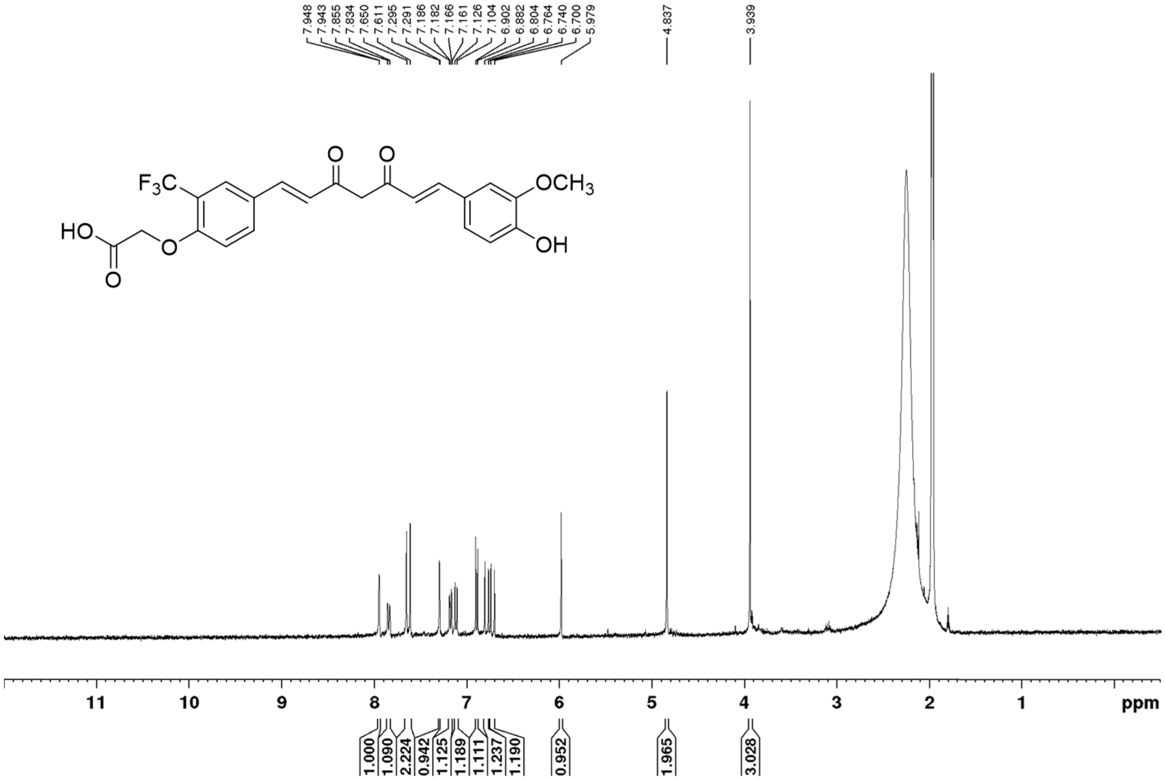

^{13}C-NMR Profile of 2-(4-((1E,6E)-7-(4-hydroxy-3-methoxyphenyl)-3,5-dioxohepta-1,6-dien-1-yl)-2-(trifluoromethyl)phenoxy)acetic acid

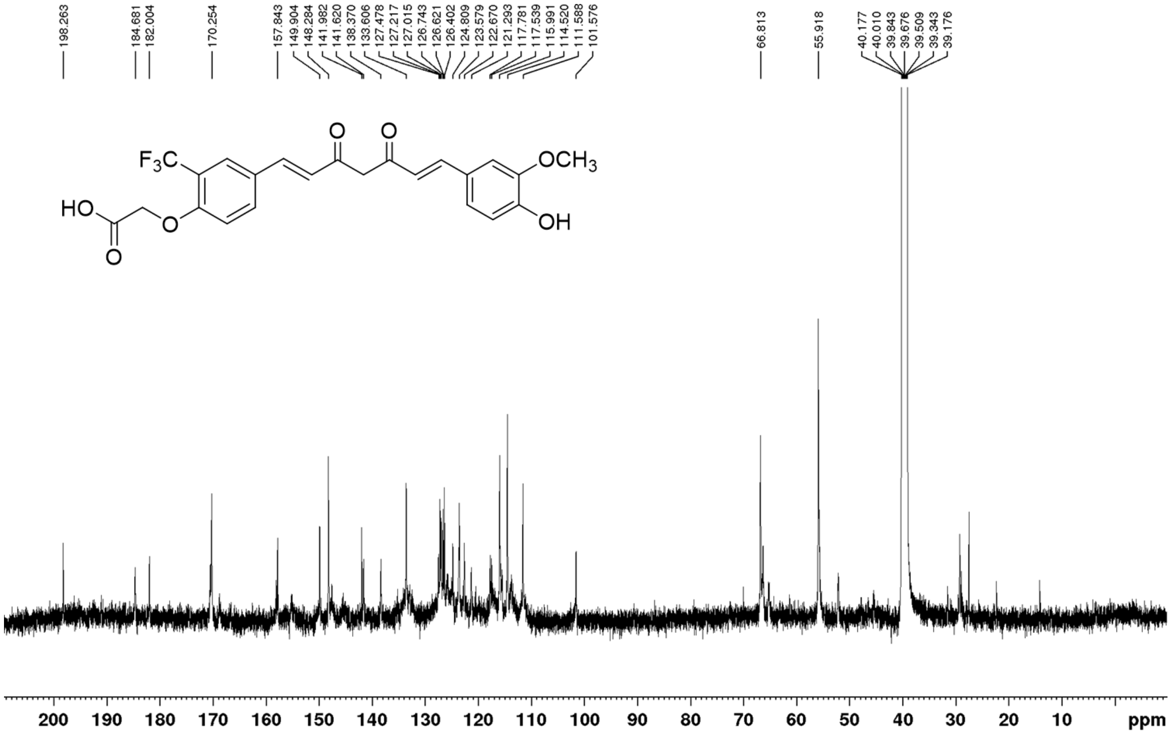

^{19}F-NMR Profile of 2-(4-((1E,6E)-7-(4-hydroxy-3-methoxyphenyl)-3,5-dioxohepta-1,6-dien-1-yl)-2-(trifluoromethyl)phenoxy)acetic acid

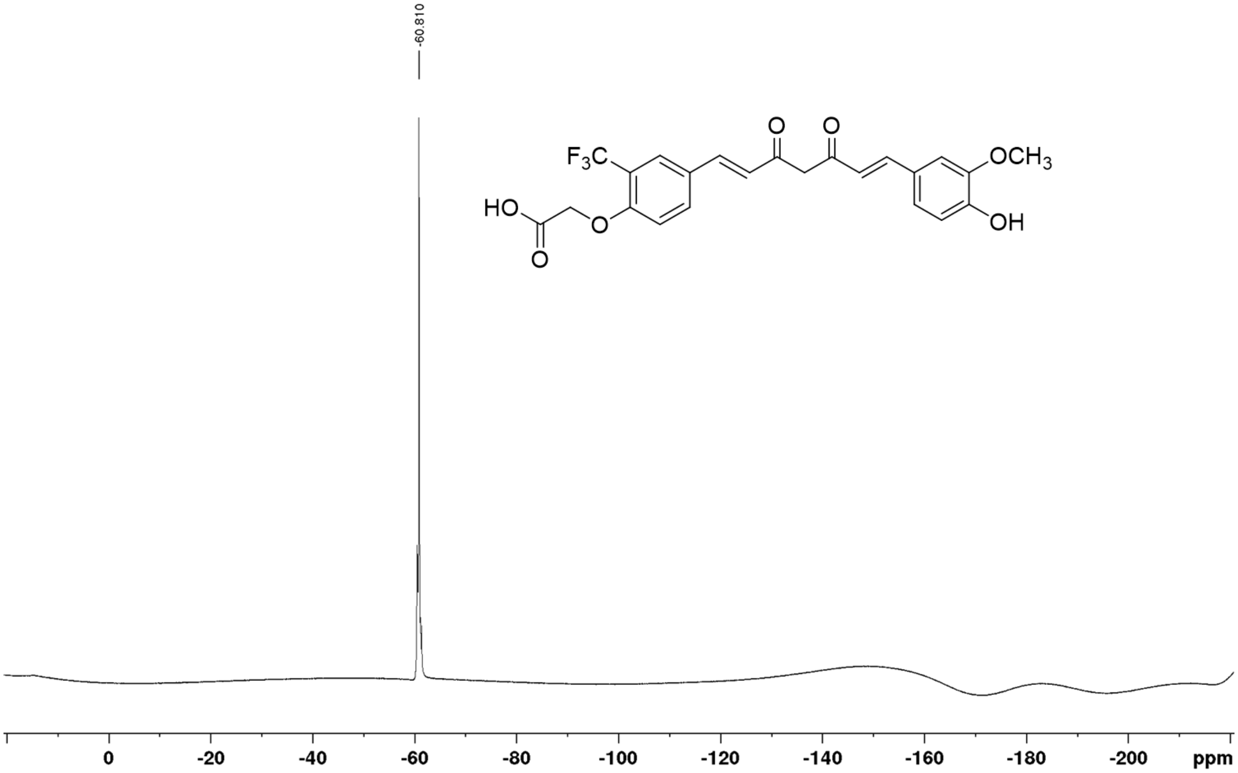

^{1}H-NMR Profile of tri-tert-butyl 2,2',2''-(10-(3-(2-(4-((1E,6E)-7-(4-hydroxy-3-methoxyphenyl)-3,5-dioxohepta-1,6-dien-1-yl)-2-methoxyphenoxy)acetamido)propyl)-1,4,7,10-tetraazacyclododecane-1,4,7-triyl)triacetate

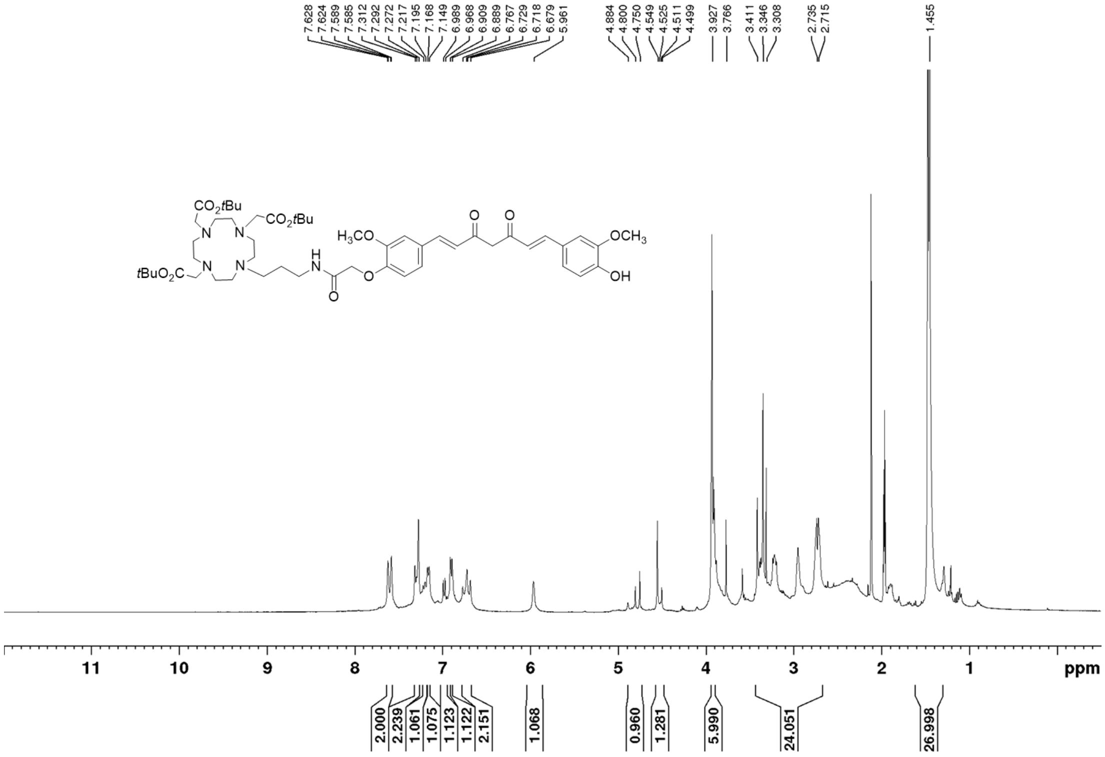

^{13}C-NMR Profile of tri-tert-butyl 2,2',2''-(10-(3-(2-(4-((1E,6E)-7-(4-hydroxy-3-methoxyphenyl)-3,5-dioxohepta-1,6-dien-1-yl)-2-methoxyphenoxy)acetamido)propyl)-1,4,7,10-tetraazacyclododecane-1,4,7-triyl)triacetate

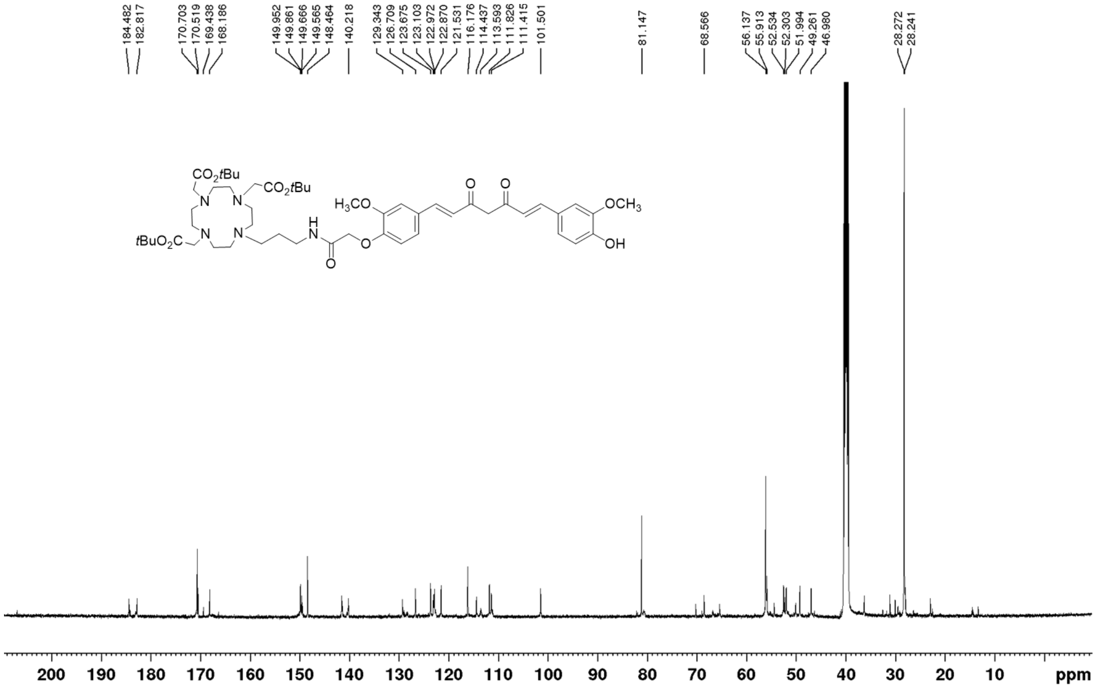

^{1}H-NMR Profile of tri-tert-butyl 2,2',2''-(10-(3-(2-(4-((1E,6E)-7-(4-hydroxy-3-methoxyphenyl)-3,5-dioxohepta-1,6-dien-1-yl)-2-(trifluoromethyl)phenoxy)acetamido)propyl)-1,4,7,10-tetraazacyclododecane-1,4,7-triyl)triacetate

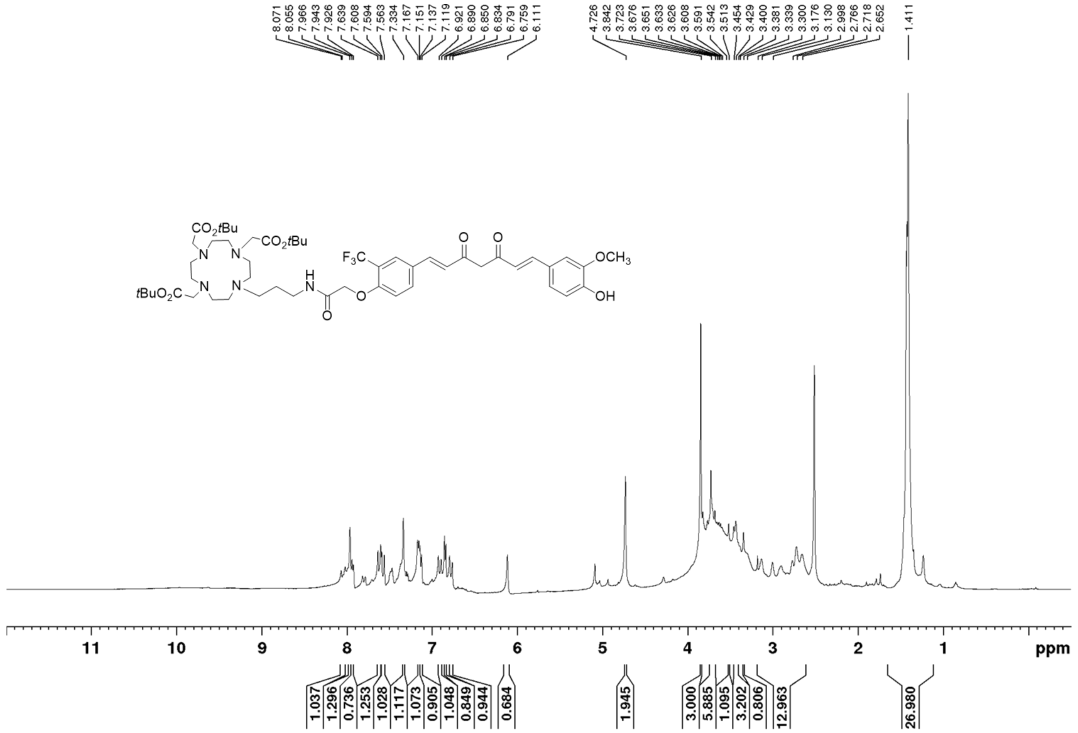

^{13}C-NMR Profile of tri-tert-butyl 2,2',2''-(10-(3-(2-(4-((1E,6E)-7-(4-hydroxy-3-methoxyphenyl)-3,5-dioxohepta-1,6-dien-1-yl)-2-(trifluoromethyl)phenoxy)acetamido)propyl)-1,4,7,10-tetraazacyclododecane-1,4,7-triyl)triacetate

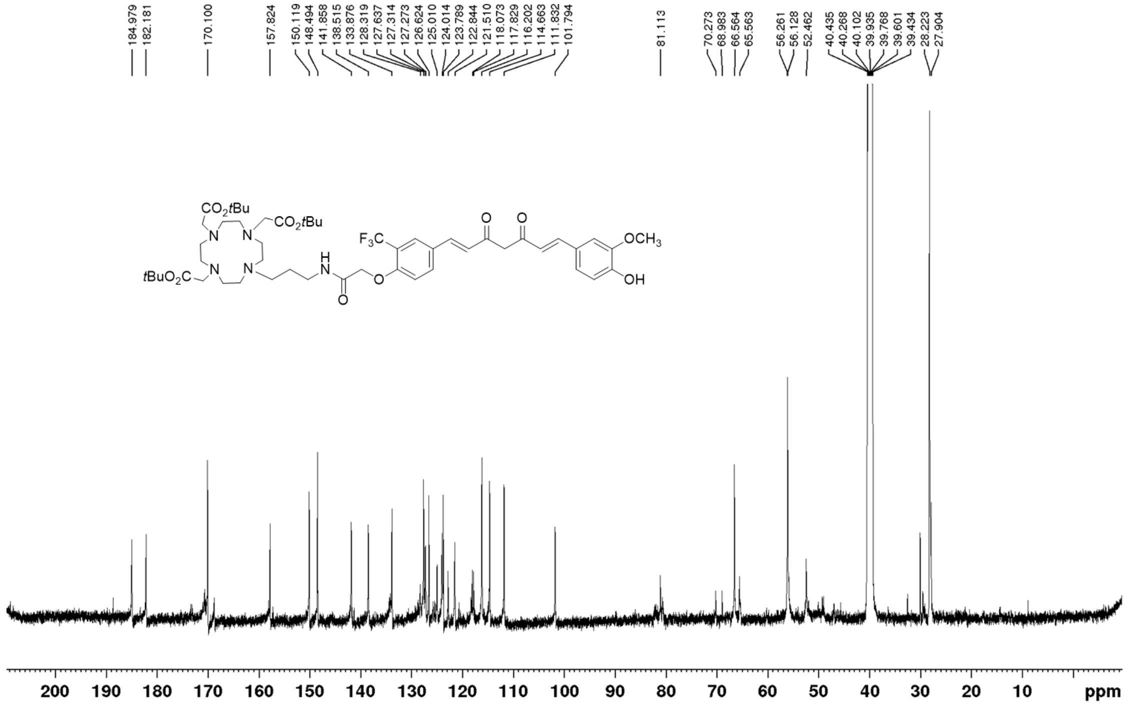

^{19}F-NMR Profile of tri-tert-butyl 2,2',2''-(10-(3-(2-(4-((1E,6E)-7-(4-hydroxy-3-methoxyphenyl)-3,5-dioxohepta-1,6-dien-1-yl)-2-(trifluoromethyl)phenoxy)acetamido)propyl)-1,4,7,10-tetraazacyclododecane-1,4,7-triyl)triacetate

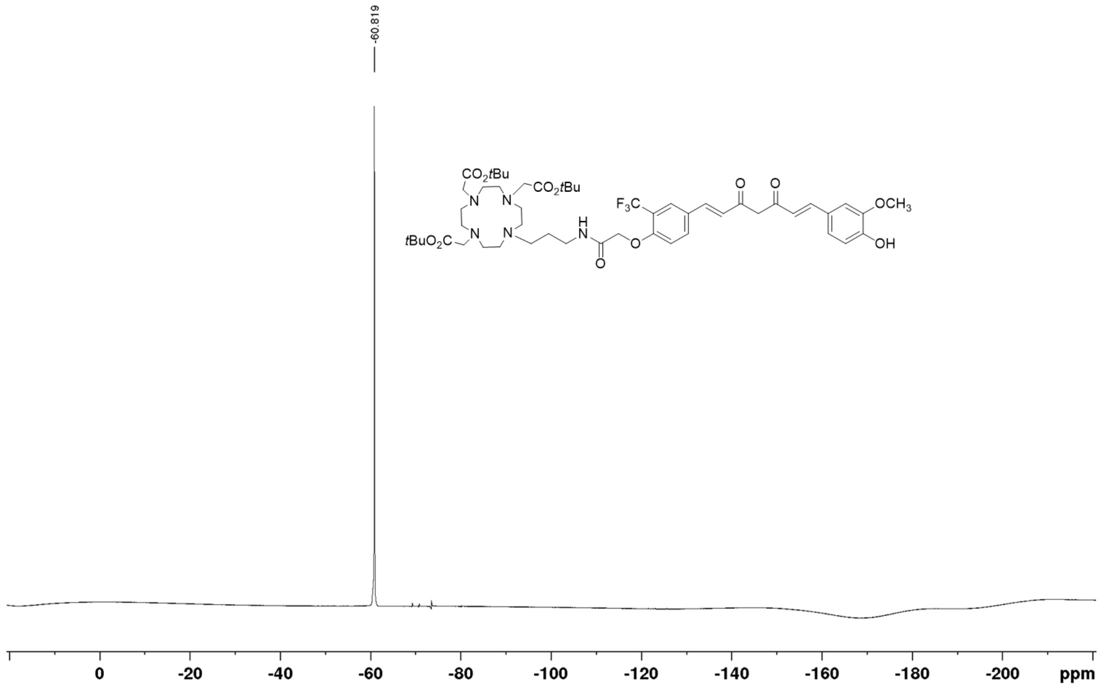

^{1}H-NMR Profile of 2,2',2''-(10-(3-(2-(4-((1E,6E)-7-(4-hydroxy-3-methoxyphenyl)-3,5-dioxohepta-1,6-dien-1-yl)-2-methoxyphenoxy)acetamido)propyl)-1,4,7,10-tetraazacyclododecane-1,4,7-triyl)triacetic acid

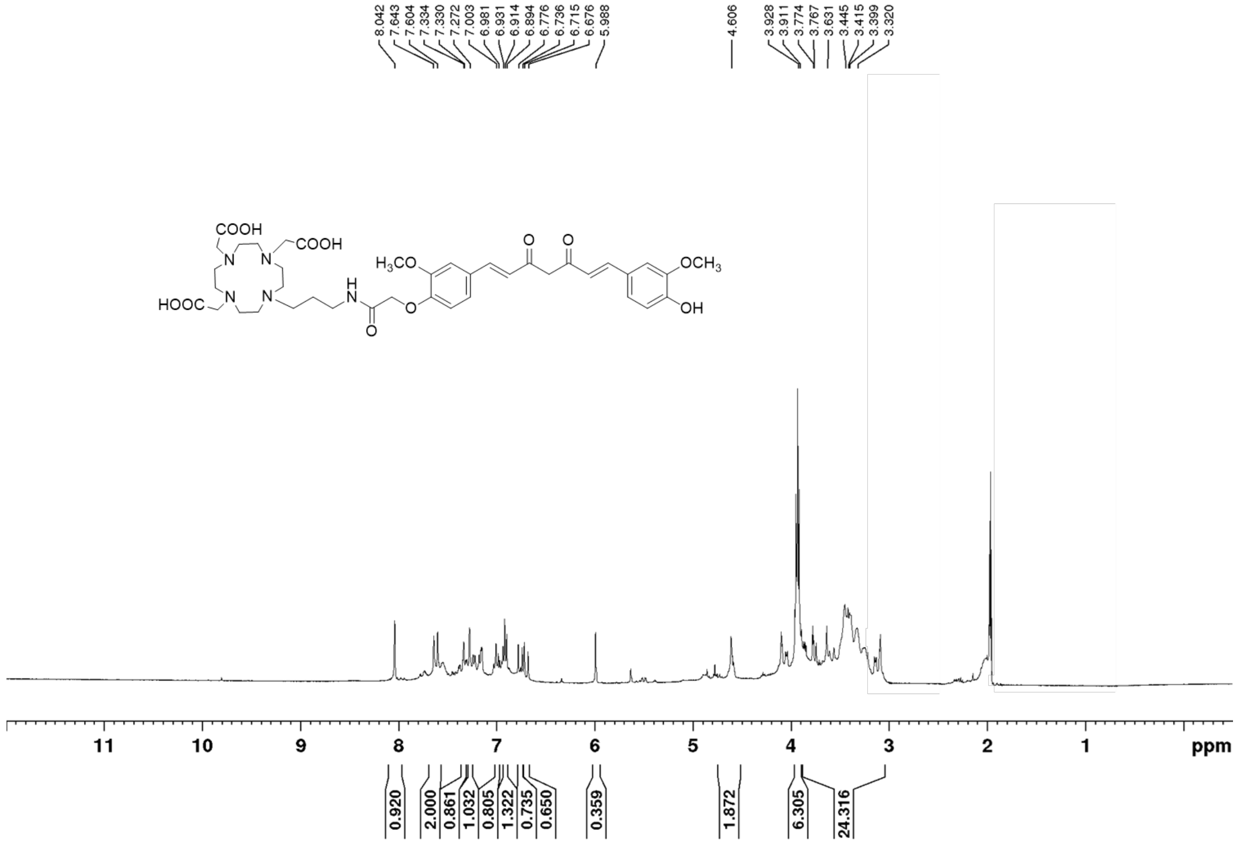

^{1}H-NMR Profile of 2,2',2''-(10-(3-(2-(4-((1E,6E)-7-(4-hydroxy-3-methoxyphenyl)-3,5-dioxohepta-1,6-dien-1-yl)-2-(trifluoromethyl)phenoxy)acetamido)propyl)-1,4,7,10-tetraazacyclododecane-1,4,7-triyl)triacetic acid

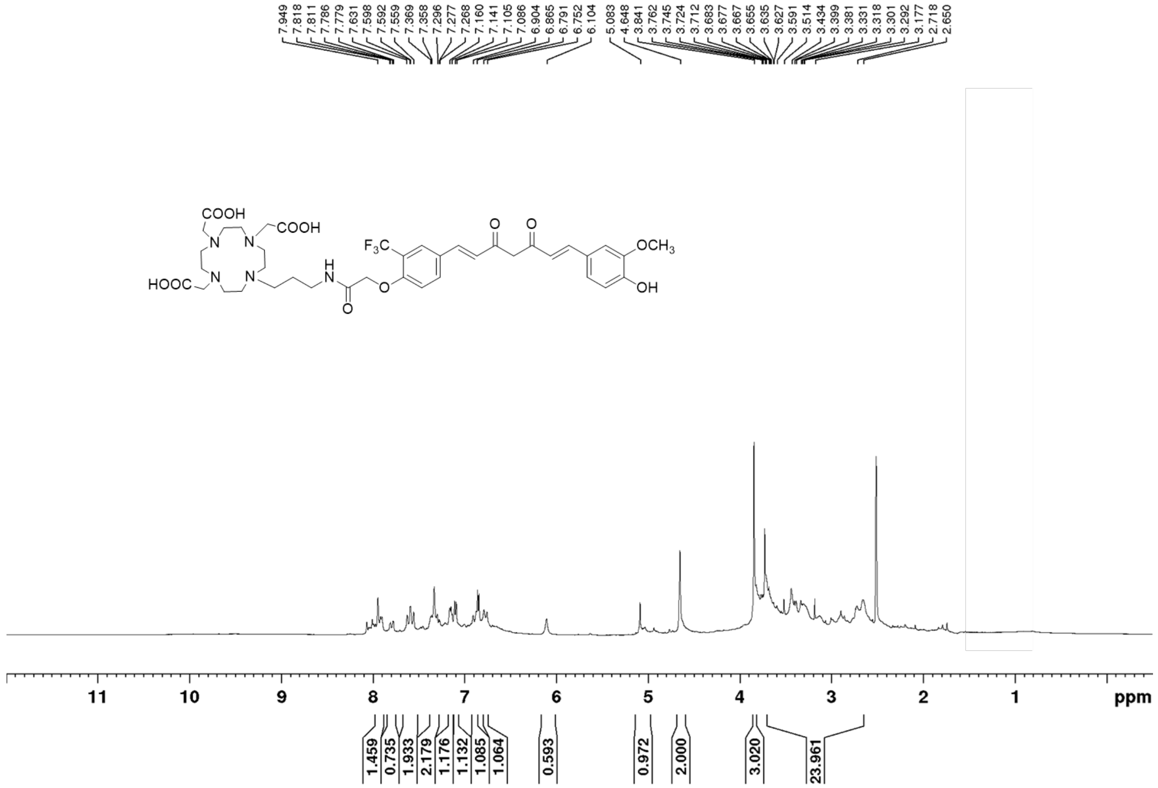

^{19}F-NMR Profile of 2,2',2''-(10-(3-(2-(4-((1E,6E)-7-(4-hydroxy-3-methoxyphenyl)-3,5-dioxohepta-1,6-dien-1-yl)-2-(trifluoromethyl)phenoxy)acetamido)propyl)-1,4,7,10-tetraazacyclododecane-1,4,7-triyl)triacetic acid

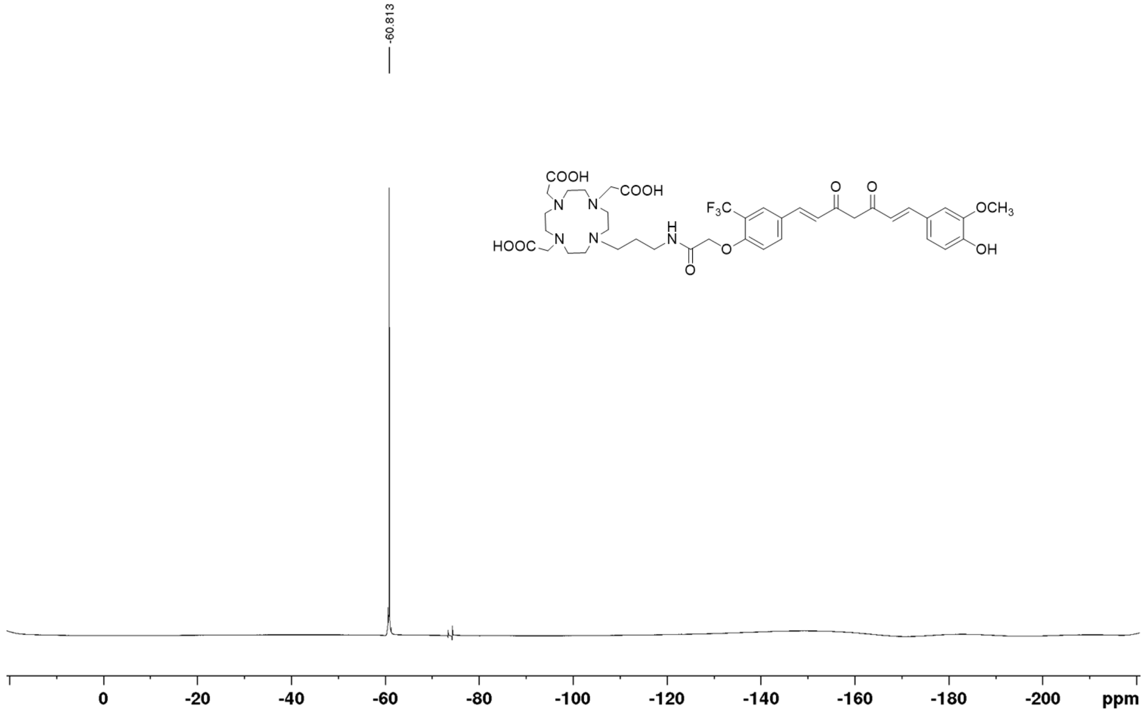

Purity Analysis of Gd-DO3A-Comp.B

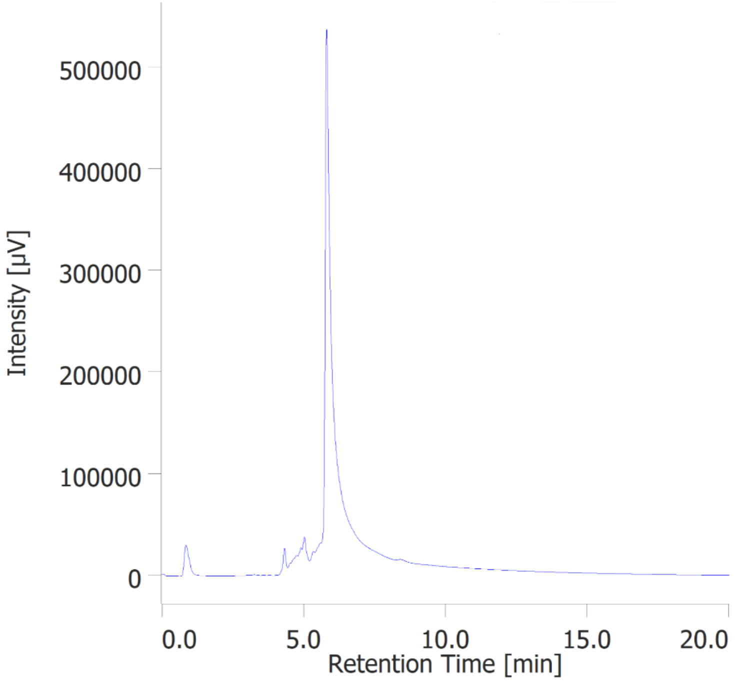

Purity Analysis of Gd-DO3A-Cur

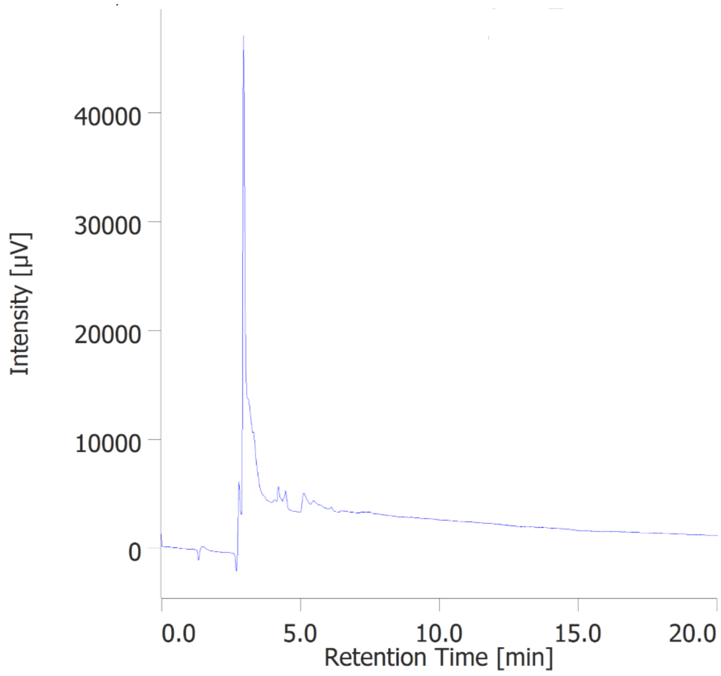

Supplement: RA-012-D2RA00614F-s001 [file RA-012-D2RA00614F-s001.pdf]
